# Secondary metabolite biosynthetic diversity in Arctic Ocean metagenomes

**DOI:** 10.1099/mgen.0.000731

**Published:** 2021-12-14

**Authors:** Adriana Rego, Antonio Fernandez-Guerra, Pedro Duarte, Philipp Assmy, Pedro N. Leão, Catarina Magalhães

**Affiliations:** ^1^​ Interdisciplinary Centre of Marine and Environmental Research (CIIMAR), University of Porto, Matosinhos, Portugal; ^2^​ Institute of Biomedical Sciences Abel Salazar (ICBAS), University of Porto, 4050-313 Porto, Portugal; ^3^​ Lundbeck Foundation GeoGenetics Centre, GLOBE Institute, University of Copenhagen, Copenhagen, Denmark; ^4^​ Norwegian Polar Institute, Fram Centre, N-9296 Tromsø, Norway; ^5^​ Faculty of Sciences, University of Porto, 4150-179 Porto, Portugal

**Keywords:** Arctic Ocean, biosynthetic gene clusters, functional metagenomics, non-ribosomal peptide synthetases, polyketide synthases

## Abstract

Polyketide synthases (PKSs) and non-ribosomal peptide synthetases (NRPSs) are mega enzymes responsible for the biosynthesis of a large fraction of natural products (NPs). Molecular markers for biosynthetic genes, such as the ketosynthase (KS) domain of PKSs, have been used to assess the diversity and distribution of biosynthetic genes in complex microbial communities. More recently, metagenomic studies have complemented and enhanced this approach by allowing the recovery of complete biosynthetic gene clusters (BGCs) from environmental DNA. In this study, the distribution and diversity of biosynthetic genes and clusters from Arctic Ocean samples (NICE-2015 expedition), was assessed using PCR-based strategies coupled with high-throughput sequencing and metagenomic analysis. In total, 149 KS domain OTU sequences were recovered, 36 % of which could not be assigned to any known BGC. In addition, 74 bacterial metagenome-assembled genomes were recovered, from which 179 BGCs were extracted. A network analysis identified potential new NP families, including non-ribosomal peptides and polyketides. Complete or near-complete BGCs were recovered, which will enable future heterologous expression efforts to uncover the respective NPs. Our study represents the first report of biosynthetic diversity assessed for Arctic Ocean metagenomes and highlights the potential of Arctic Ocean planktonic microbiomes for the discovery of novel secondary metabolites. The strategy employed in this study will enable future bioprospection, by identifying promising samples for bacterial isolation efforts, while providing also full-length BGCs for heterologous expression.

## Data Summary

Metagenomic datasets are available at European Nucleotide Archive under the project accession number PRJEB15043.

**Impact Statement**

Marine microorganisms are widely known as a rich source of natural products and the marine environment harbours an unexplored potential for the discovery of new chemical diversity. Polar marine environments have been particularly overlooked and the biosynthetic potential of planktonic microbial communities from the Arctic Ocean has not yet been a target of study using metagenomics approaches. In this study, a total of 149 KS domain OTU sequences were recovered, 36 % of which could not be assigned to any known BGC. In addition, 74 bacterial metagenome-assembled genomes were recovered, from which 179 BGCs were extracted. The majority of the recovered BGCs are distantly related to MIBiG BGCs, thus likely to be involved in the production of novel compounds, highlighting the potential of Arctic Ocean planktonic microbiomes for the discovery of novel secondary metabolites.

## Introduction

Marine microorganisms represent a prolific source of bioactive natural products (NPs) which have the potential to become drug leads [1]. Among the several structural/biosynthetic classes of NPs [2], polyketides (PKs) and non-ribosomal peptides (NRPs) make up for a large number of compounds that have reached clinical application [3]. PKs and NRPs are generated by polyketide synthase (PKS) and non-ribosomal peptide synthetase (NRPS) enzymatic assembly lines, respectively. Ribosomally synthesized and post-translationally modified peptides (RiPPs) are another important class of bioactive secondary metabolites that have recently garnered attention as they can be easily identified from genome data-derived structural predictions [3].

With the recent advances in nucleic acid sequencing technologies and bioinformatics, the biosynthetic richness of environmental microbiomes and uncultured bacteria has been brought to light [4, 5]. Molecular markers targeting biosynthetic genes, such as the ketosyntase (KS) domain of PKSs, have been used to assess the diversity and distribution of biosynthetic genes of bacterial isolates and complex microbial communities [6, 7]. Functional metagenomics, which aims at recovering functional genes, such as those found in BGCs, from metagenomes and expressing them in culturable organisms [8], provides a path to unveil new natural products from uncultured bacteria [9]. The use of metagenomic libraries for screening of sequence tags, such as KS domain of PKSs and for screening of phenotypes, such as antibiotic or enzyme production [10, 11] allowed for the identification of genes classes of unknown functions and identification of new compounds [12]. More recently, metagenomics approaches have facilitated the recovery of complete biosynthetic gene clusters (BGCs) from environmental DNA.

Despite the fact that the ocean covers over 70% of the Earth’s surface, the marine environment is still considered one of the most understudied ecosystems [13]. This applies particularly to marine polar environments, which are difficult to access due to the extreme environmental conditions [14]. The harsh conditions in the polar areas, with sub-zero temperatures and extreme day-night cycles, have led to unique functional diversity, usually associated with modifications in gene regulation and metabolic pathways necessary to adapt to polar environments [15]. The diversity found in polar organisms translates also into an increased chance of discovery of novel enzymes and NPs [15]. Despite this, NPs derived from polar organisms represent only 3 % of all the described natural products [16], in part due to the difficulty to cultivate polar bacteria in the laboratory [17] and the inaccessibility of these remote environments. Recent studies have highlighted the untapped NPs discovery potential of polar bacteria, namely PKs and NRPs [14, 18]. Some studies [19–21] have looked into Arctic marine sediments for the associated genetic potential to encode NPs, and found new esterases and deacetylase enzymes. However, to the best of our knowledge, Arctic planktonic microbial communities have not been a target of metagenomics-based bioprospection.

In this study, we use both amplicon sequencing and shotgun metagenomics to characterize the potential of Arctic Ocean planktonic communities for NPs production.

## Methods

### Sampling and sequencing

Samples used in this study were collected during the Norwegian young sea ICE (N-ICE 2015) expedition in the Arctic Ocean north of Svalbard, as described in elsewhere [22–25]. Seawater samples were collected at surface (5 m), subsurface (20 or 50 m), and mesopelagic (250 m) depths across three distinct sites, the deep Nansen Basin (NB), the shallower Yermak Plateau (YP) and the slope towards YP, the Transition Region (TR) (Table 1).

**Table 1.** Environmental parameters described for the samples in study including water depth, sampling site, season, latitude and longitude

Sample ID	NB_5	NB_50	NB_250	TR_50	TR_250	YP_20	Reference
Water depth (m)	5	50	250	50	250	20	[25]
Sampling site	Nansen Basin	Nansen Basin	Nansen Basin	Transition region	Transition region	Yermak Plateau	[25]
Season	Late Winter	Late winter	Late winter	Early Spring	Early Spring	Early Summer	[25]
Latitude	83° 10.002´N	83° 10.002´N	83° 10.002´N	82° 23.195´N	82° 23.195´N	80° 30.775´N	[25]
Longitude	22° 01.998’E	22° 01.998’E	22° 01.998’E	15° 9.198´E	15° 9.198´E	07° 52.428´E	[25]

DNA extraction and sequencing are described in detail in Sousa *et al*. [25]. Briefly, DNA was extracted from SterivexTM filters using PowerWater DNA Isolation Kit protocol (MO BIO Laboratories, Inc.) and shotgun metagenomic sequencing was performed in Illumina MiSeq sequencer using V3 Chemistry (Illumina), by LGC Genomics (LGC Genomics GmbH, Berlin, Germany).

For amplification of KS domains from PKS genes, degenerate primer pairs degKS2F (5′ GCNATGGAYCCNCARCARMGNVT)/degKS2R(5′GTNCCNGTNCCRTGNSCYTCNAC) [26] were used. The PCR reaction was prepared in a volume of 20 µl containing 1×TaKaRA PCR Buffer (TAKARA BIO INC, Shiga, Japan), 1.5 mM MgCl2 (TAKARA BIO INC, Shiga, Japan), 250 µM dNTPs (TAKARA BIO INC, Shiga, Japan), 0.625 µl of each primer (100 µM), 0.25 mg ml^−1^ of UltraPureTM BSA (Life technologies, Waltham, MA USA), 0.5 U TaKaRa Taq Hot Start Version (TAKARA BIO INC Shiga, Japan), and 2 µl of template DNA. The PCR conditions were as follows: initial denaturation step at 95 °C for 4 min, followed by 40 cycles of a denaturation step at 94 °C during 40 s, annealing at 56.3 °C for 40 s (KS), and extension at 72 °C for 75 s, followed by a final extension step at 72 °C for 5 min. Amplified PCR products were sequenced using Illumina MiSeq 2×300 technology, at LGC Genomics (LGC Genomics GmbH, Berlin, Germany).

### Metagenome pre-processing and quality checking for BiG-MEx analysis

The analysis was performed on metagenomic data with adapter-clipped files. The paired-end reads were merged using the ‘fastq_mergepairs’ command in VSEARCH v2.7.0 [27]. Both merged and unmerged reads were quality trimmed at Q20 and sequences shorter than 45 bp were removed using the BBDuk tool from BBMap (https://sourceforge.net/projects/bbmap/). Singleton reads that have passed the filter when their mate failed were also retained, using ‘outs’ (outsingle) parameter. All the output files (quality filtered merged and unmerged reads as well as singleton reads) were concatenated in a single file that was then used for the dereplication using the ‘vsearch--derep_fulllength’ command. The de-replicated file was then used for the ORF prediction using FragGeneScan-plus [28].

Processed files and predicted open reading frames (ORFs) were run in BiG-MEx [29], a tool for mining BGC domains and classes in metagenomic data. This analysis allows the identification of BGC protein domains, compute BGC domain-based diversity analysis and compute BGC class abundance predictions [30]. The predicted ORF amino acid sequences were used to run the BGC domain annotation. For determining BGC domain diversity, the unmerged and pre-processed data was used together with the UProc output generated during the module BGC domain annotation (se_bgc_dom.gz). After computing the BGC domain diversity for each sample, the merge mode of the ‘bgc_dom_div’ command was used and a reference phylogenetic tree including the placed domain sequences from all samples was obtained for PKS-KS domain of PKS and for condensation domain of NRPS. Briefly, as described by Pereira-Flores *et al*. [29], the phylogenetic placement consists of aligning the domain sequences to their corresponding reference multiple sequence alignment (MSA) and the extended MSA together with its reference tree are then used as the input to run pplacer [30], which performs the phylogenetic placement using the maximum-likelihood criterium and outputs the extended tree.

For the BGC class prediction, the output ‘counts.tbl’ generated during the BGC domain annotation was used. For computing pruned trees, the newick file generated by BiG-MEx was edited using iTOL [31]. An additional analysis was performed for the functional annotation of KS domain sequences, so as to allow the comparison with the amplicon-derived data. KS domain OTU sequences recovered from BiG-MEx analysis were aligned locally with BLAST + version 2.9.0 against the MIBiG [32] database v.1.4. blast matches with an e-value larger than 10^−20^ are reported as Not Assigned (NA).

### Sequence analysis of KS domains

Primer-clipped forward and reverse fastq sequences from KS domains were quality trimmed using bbduk and truncated to 240 and 175 bp, respectively, using USEARCH v11.0.667 [33]. The reads were then reordered to obtain the correct match pairs (using the repair.sh tool from BBMap, available online: https://sourceforge.net/projects/bbmap/) and the matching pairs were concatenated with an intervening ‘N’ using USEARCH. The sequence identifiers of each sample were renamed to allow for discrimination on the statistical analysis and all the samples were combined in a single file. The sequences were dereplicated using USEARCH, clustered at 97% identity, the singletons were removed, and a second round of clustering at 95% of identity was performed, as described previously [34]. Finally, VSEARCH v2.10.4 [27] was used to generate an OTU (operational taxonomical unit) table that was imported to phyloseq [35] for alpha and beta-diversity analysis. The alpha-diversity metrics computed were the number of observed OTUs and Shannon; for beta-diversity, the Bray–Curtis metric was estimated and visualized through principal coordinate analysis method (PCoA). The final plots were obtained using the ggplot2 v.3.1.0 [36] R package.

For the functional annotation of KS domain sequences, OTU sequences were aligned locally with BLAST + version 2.9.0 against the MIBiG [32] database v.1.4. blast matches with an e-value above 10^−20^ are reported as Not Assigned (NA).

### Metagenome pre-processing and quality checking for anti-SMASH analysis

BiosyntheticSPAdes v.3.14 [37] was used to assemble metagenomes to be used in antiSMASH [38]. Adapter-clipped paired-end reads were quality trimmed at Q20 and sequences shorter than 45 bp were removed using BBDuk tool from BBMap (https://sourceforge.net/projects/bbmap/). Singleton reads that have passed the filter when their mate failed were also retained, using ‘outs’ (outsingle) parameter. Both paired-end and singleton reads were used for the assembling. DNA fasta files of assembled candidate BGCs were submitted to a locally installed version of antiSMASH (v. 5.1.2).

### Metagenome-assembled genomes (MAGs)

For the recovery of metagenome assembled genomes (MAGs), paired-end quality filtered reads were co-assembled using MEGAHIT v.1.2.9 [39]. Mapping was performed using Bowtie2 v.2.3.5.1 [40, 41] and samtools v.1.10 [42] and the following steps were preformed using Anvi’o v.6.1 [43] following the tutorial ‘Metagenomic workflow’ (http://merenlab.org/2016/06/22/anvio-tutorial-v2/). Briefly, a contigs database was created using ‘anvi-gen-contigs-database’, open reading frames were identified using Prodigal [44] and genes in the contigs matching bacterial single-copy core genes were identified using HMMER [45]. For functional annotation of genes in the contigs database, ‘anvi-run-ncbi-cogs’ was used. Profiling was performed using ‘anvi-profile’ and obtained Anvi’o profiles merged using ‘anvi-merge’. Binning was performed using the standalone tool CONCOCT v.1.1.0 [46] and the generated bins were imported to Anvi’o using ‘anvi-import-collection’. Taxonomy was estimated using ‘anvi-estimate-genome-taxonomy’. To visualize the bins, ‘anvi-interactive’ was used and bins were manually refined using ‘anvi-refine’. The final taxonomy was assigned to the refined bins using GTDB-Tk v0.3.3 [47]. Quality of the recovered MAGs was determined using Anvi’o ‘anvi-estimate-genome-completeness’. Refined bins were considered MAGS of high-quality if they had >90% completeness and <5% contamination and medium quality with >50% completion and <10% contamination. Recovered bins with completion lower than 50 % or contamination >10% were excluded.

### Phylogenomic analysis

Phylogenomic trees were computed using PhyloPhlAn 3.0 [48]. PhyloPhlAn uses 400 universal marker genes to resolve phylogenetic trees of high-diversity genomes. Maximum-likelihood trees using LG substitution model were obtained. A phylogenomic tree of all the recovered MAGs was computed, and to understand the novelty of the recovered MAGs, order- or genus-specific phylogenomic trees were computed for MAGs that had public genomes available. The resulting phylogenomic trees were imported and edited using iTOL [31].

### BiG-SCAPE sequence similarity networks

Obtained MAGs were run in antiSMASH v.5.1.2. A network analysis of recovered BGCs was performed using the BiG-SCAPE [49] algorithm. The programme was run in the default global mode. The networks were computed using the ‘mix’ and ‘mibig’ options, which include an analysis mixing all classes and includes MIBiG BGCs in the network. The option ‘--include singletons’ was also used, to allow visualizing singleton BGCs in the network. Networks were computed using multiple raw distance cutoff values (from 0.1 to 1.0) and the networks computed using 0.7 cutoff were chosen. According to BiG-SCAPE instructions [49] tighter (lower) cutoffs are more appropriate for grouping BGCs that produce identical compounds while looser (higher) cutoffs provide a broader perspective on related NP families. The resulting sequence similarity matrices were then visualized in Cytoscape v.3.7.2 [50] and a column with the assignment of each BGC to the category MIBiG or MAG was included to differentiate between the two sets of BGCs.

## Results and discussion

### Biosynthetic diversity assessed through metagenomes

Biosynthetic diversity was directly assessed from six metagenomes generated from Arctic Ocean microbial samples. The number of unique sequences after dereplication, from the whole data set, varied between 2 902 598 (sample TR_250) and 4 146 285 (sample NB_50) (Table S1, available in the online version of this article), and the number of predicted ORFs between 2 975 689 (sample TR_250) and 4 226 932 (sample NB_50) (Table S1). Predicted ORFs and pre-processed reads (Table S1) were used in BiG-MEx annotation and diversity modules. BiG-MEx analysis revealed that the metagenomes analysed are mostly rich in genes associated with NRPs, lanthipeptides, saccharides and fatty acids (Table 2).

**Table 2.** Abundance of the dominant domains (abundance >800) and respective BGC classes across the different samples

BGC class	Domain	NB_5	NB_50	NB_250	TR_50	TR_250	YP_20
NRPS	AMP.binding	9468	11337	12316	11786	13424	17300
Lanthipeptide	PF00106	2310	2679	4287	2947	3423	3979
Lanthipeptide	PF13561	2036	2339	1574	2056	1729	3052
Oligosaccharide	Glycos_transf_2	1599	1976	950	1340	1123	1415
Lanthipeptide	PF00067	1256	1872	997	1165	856	1726
other	NAD_binding_4	1175	1458	1300	1108	983	1711
Fatty acid	t2fas	1096	1129	1128	1059	856	1354
Saccharide	Glycos_transf_1	960	1168	1114	949	832	1243

NRPs are, together with PKs, associated with a broad range of pharmacological properties [51]. Lanthipeptides are polycyclic RiPP [52] NPs, often with antibiotic properties [53]. Both saccharides and fatty-acids play important ecological roles in cold environments, including in the Arctic [54]. Interestingly, in a study by Benaud *et al*. [55], using amplification of PKS and NRPS genes in desert polar soils, which included samples from Svalbard, NRPS sequences were not retrieved. In a recent study developed by our team using amplification of PKS (KS domain) and NRPS (AD domain) genes, from maritime samples from Antarctica, the number of KS domain OTUs recovered was significantly higher than for AD domains [5]. On the other hand, several studies targeting marine environments [56, 57], recorded a higher abundance of NRPS OTU sequences when compared to those of PKSs, which is in agreement with our results. Regarding BGC domain abundance, sample YP_20 was found to harbour a higher abundance of the different domains compared to the remaining samples (Table 2), being particularly enriched in NRPS. This was the only sample collected in early summer (June 16) during an under-ice bloom of the haptophyte algae *Phaeocystis pouchetii* [22]. Besides, sample YP_20 was the only in this study to be collected over the YP, a local hotspot for vertical mixing and cooling of Atlantic water [24], where higher salinity, oxygen and PAR levels were registered [25]. Previous studies have revealed that, for example, actinobacteria richness [58], geographic location, latitude and pH [59] can impact biosynthetic diversity in environmental microbiomes. In our samples, a PCA analysis (Fig. S1) revealed that sample YP_20 groups independently from the remaining samples according to environmental factors, highlighting their potential influence on biosynthetic diversity.

**Fig. 1.**
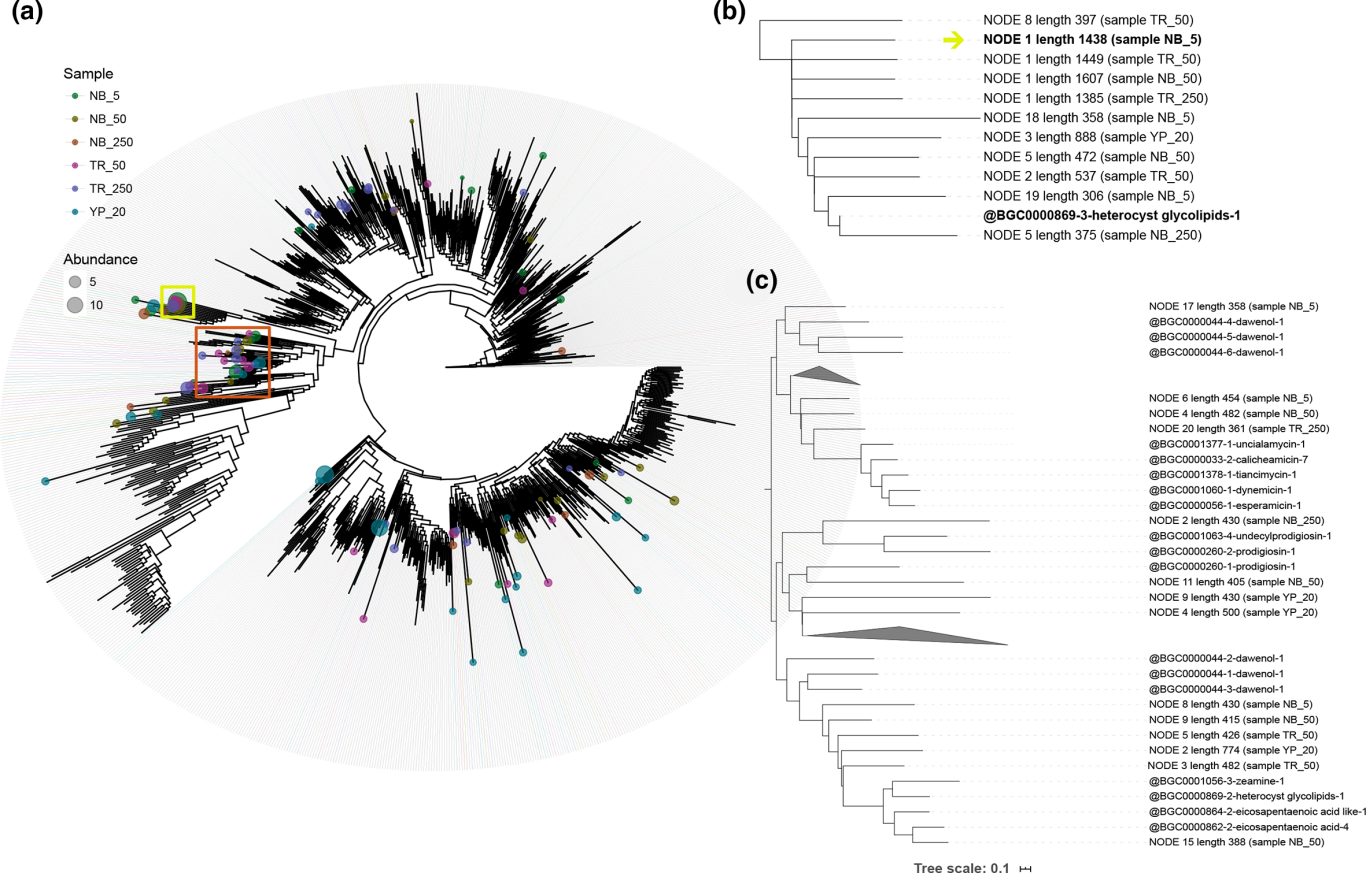
(a) Placement of recovered PKS-KS domain representative OTU sequences onto a pre-computed reference phylogenetic tree including PKS-KS domain sequences from MIBiG database, performed by the BiG-MEx domain-based diversity analysis module. In the phylogenetic tree, the size of the bubbles on the leaves represents the abundance of the recovered KS representative OTUs sequences and the colour represents the sample from it originates. For a detailed inspection and identification of closest domains of BGCs from MIBiG database, the clades harbouring the most abundant OTU (NODE one from sample NB_5), highlighted by a yellow square, and a large clade containing several representative OTUs sequences (highlighted by an orange square) were selected to compute the pruned trees b and c, respectively. (**b**) Partial tree of the clade harbouring the most abundant OTU, indicated by the yellow arrow. (**c**) Partial tree of a clade containing several representative OTU sequences, indicated by an orange square on tree a.

Bioprospection in Arctic microorganisms focuses mostly on cold-adapted enzymes [60] such as esterases [19, 61] and α-amylases [62]. Novel NPs recovered from Arctic bacteria include a few cytotoxic and antiviral molecules, such as mixirins [63] and nitrosporeusines [64], respectively. Fatty-acids, particularly polyunsaturated fatty acids (PUFAs), due to their role in cold adaptation [65] are also commonly reported in polar bacteria and algae [66]. The phylogenetic distribution of the recovered PKS-KS domain sequences and their association to metabolites using the MIBiG database (Fig. 1) indicates that several sequences retrieved in this study are closely related to the red pigment prodigiosin [67], the fatty-acid eicosapentaenoic acid (EPA) and the PUFA-like PK dawenol [68]. While fatty-acids are usually involved in cold adaptation [65], pigments are involved in UV, desiccation and extreme temperatures protection [54], and can also act as antimicrobial agents [69]. The most abundant KS domain sequence recovered, obtained from sample NB_5 (the one collected closest to surface), is closely associated to heterocyst glycolipids, cyanobacterial metabolites that act as a gas barrier in these N_2_-fixing specialized cells [70].

**Fig. 2.**
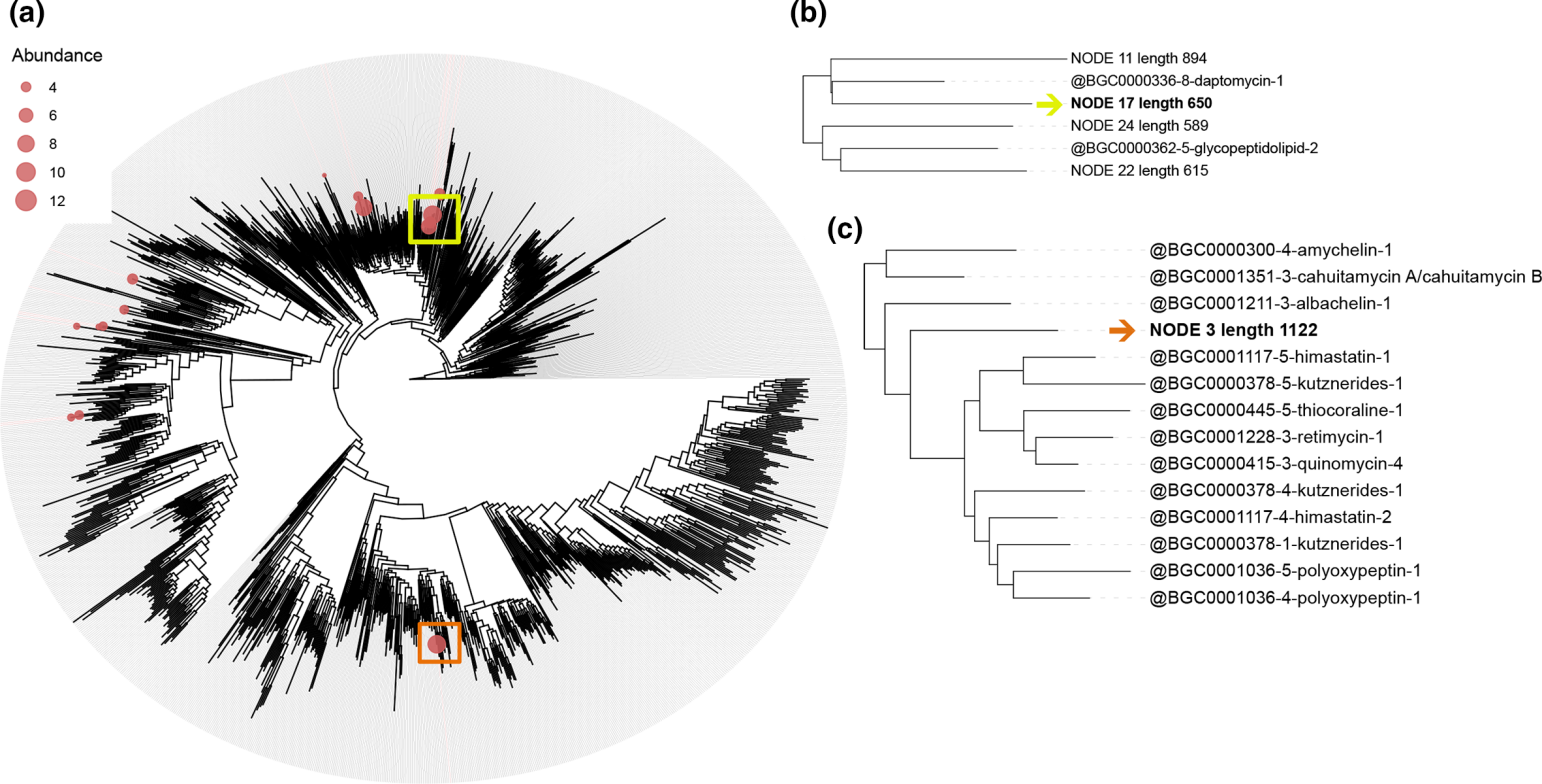
(a) Placement of recovered NRPS condensation domain representative OTU sequences from sample YP_20 onto a pre-computed reference phylogenetic tree including NRPS condensation domain sequences from MIBiG database, performed by the BiG-MEx domain-based diversity analysis module. In the phylogenetic tree, the size of the bubbles on the leaves represents the abundance of the recovered representative OTUs sequences. The clades harbouring the most abundant OTU (NODE 17), highlighted by a yellow square, and a clade that contains an OTU (NODE 3) not associated with metabolites from MIBiG database (highlighted by an orange square) were selected to compute the pruned trees b and c, respectively. (**b**) Partial tree of the clade harbouring the most abundant OTU, indicated by the yellow arrow. (**c**) Partial tree of a clade containing an OTU (indicated by the orange arrow) not closely associated with NRPS condensation domains from MIBiG database.

For the sample with a higher richness in NRPSs (sample YP_20), it was possible to perform a phylogenetic analysis of the associated condensation domains (Fig. 2), which revealed that the most abundant representative sequences were closely associated to the antibiotics daptomycin [71] and salinamide [72], both isolated from marine *Streptomyces* species, and to the siderophore fuscachelin [73]. Further, it was possible to distinguish some sequences not associated with metabolites in MIBiG (such as NODE three in Fig. 2), therefore representing domains potentially involved in the biosynthesis of highly novel metabolites.

PKS sequences were detected in low abundance from the obtained metagenomes (Table S2). To recover a higher diversity of PKS, a PCR-based approach, through amplification of KS domain, was used. This strategy has been previously used by our team to study the biosynthetic diversity of maritime samples from Antarctica [5]. In total, 149 KS domain OTUs were successfully recovered, compared to the metagenome-derived 106 KS OTUs that had been previously recovered (Fig. S2). Alpha-diversity indices (Observed OTUs and Shannon) indicated that samples NB_250 and TR_250 were the most biosynthetically diverse (Fig. S3). As found in the metagenome analysis, sample YP_20 showed a distinct pattern compared to the other samples and did not cluster with any other sample in the dataset, according to the diversity indices (Fig. S3), what might be explained due to the presence of bacteria associated to the bloom of the haptophyte algae *Phaeocystis pouchetii* [22].

KS domain OTUs were matched by blast against MIBiG, to infer whether these were associated with known BGCs or rather would be indicative of novel chemical diversity. The OTU with a highest match to MIBiG database (Table S3), showed nearly 90% identity to the BGC of phenylnannolone A, a multidrug resistance reversal agent isolated from the proteobacterium *Nannocystis pusilla* B150 [74]. About 36% of the OTUs could not be assigned to any known metabolite and sample YP_20 contained the highest number of OTUs without a match to the database, highlighting its potential for the discovery of new polyketide natural products. In our previous study, using maritime samples from Antarctica, a total of 210 KS domain OTUs were recovered and the percentage of unassigned OTUs corresponded to 11% [5]. Altogether these results highlight the unexplored chemical diversity present in polar areas.

### BGCs from assembled metagenomes

In addition to the information on biosynthetic diversity that was obtained through the retrieval of biosynthetic genes or conserved domains, we sought to recover extended BGC sequence space from our samples, which could in turn lead to the isolation of novel compounds through heterologous expression [3]. In order to recover complete or near-complete BGCs, assembled metagenomes were first obtained. Metagenomes were assembled (Table S4) and DNA sequences from putative BGCs recovered using BiosyntheticSPAdes [37]. BiosyntheticSPAdes was able to assemble PKS and NRPS BGCs, but the number of BGCs recovered was relatively low (Table 3). A higher number of BGCs was recovered from sample YP_20 and TR_250 (Table 3). From sample YP_20, in particular, we recovered several complete or near-complete BGCs (Table 3), including a Type III PKS of over 40 kb and a NRPS BGC with over 97 kb (Fig. S4). According to the most similar known cluster, a Type I PKS was identified with 75% identity to branched-chain fatty acids and a NRPS-like with 20% identity to indigoidine [75].

**Table 3.** Statistics obtained using BiosyntheticSPAdes, results correspond to quality filtered R1 and R2 input files, together with singleton reads. Number of candidate BGCs recovered for each sample and distribution of domains (biosyntheticSPADES) and antiSMASH results, including the maximum and minimum length of recovered BGCs

		Domains						antiSMASH		
Samples	Candidate BGCs (nr)	AMP	AT	C	KR	KS	TE	Clusters identified (nr)	Max. lenght (bp)	Min. length (bp)
NB_5	134	1129	215	26	1025	238	66	3	29216	2157
NB_50	152	1362	210	49	1162	244	76	2	22273	1415
NB_250	106	748	107	16	782	113	44	1	15312	15 312
TR50	186	1329	168	47	1321	225	78	5	6423	1009
TR250	226	1483	160	38	1412	196	54	6	16257	1350
YP20	295	1639	196	169	1297	328	105	68	97343	1965

Candidate clusters recovered using biosyntheticSPADes were also analysed by blast against the MIBiG database. From these, it was possible to identify a few high identity matches (Table S5), namely to polyhydroxyalkanoate [76], to the saccharide aminoglycoside istamycin [77] and to the pigment flexirubin [78], that were not detected via antiSMASH analysis.

### BGCS from metagenome assembled genomes

**Fig. 3.**
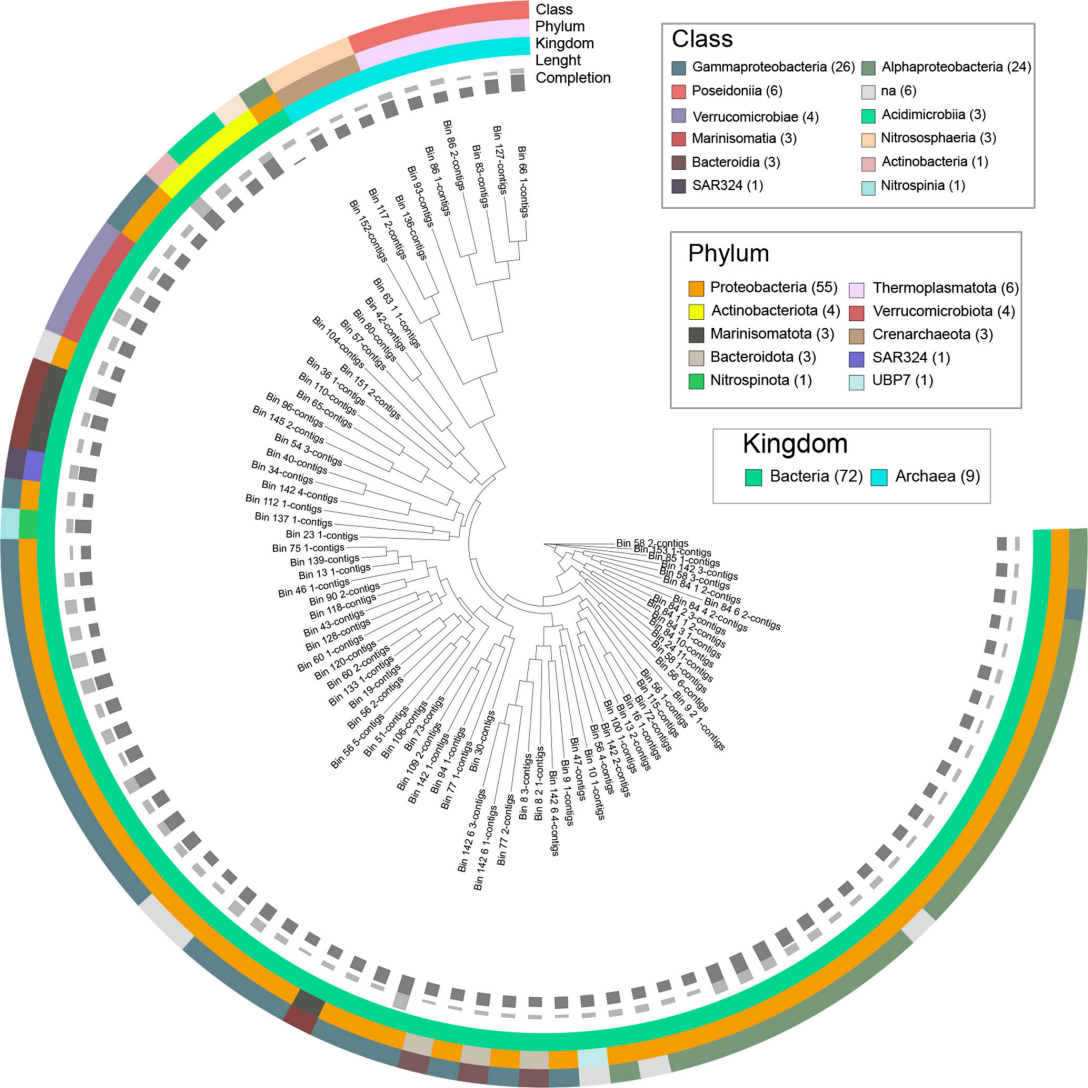
Phylogenomic tree of the MAGs recovered in this study. Information of completion (bar represented from 50.7–100 % of completion), total length (bar represented from 693818 to 5878766 nt) and the Kingdom, Phylum and Class associated to each MAG is included. The Maximum-likelihood phylogenomic tree was computed using Phylophlan and edited using iTOL.

To increase the number of complete or near-complete recovered BGCs, metagenome assembled genomes (MAGs) were obtained. After refinement, a total of 246 Bins were recovered, from which 11 corresponded to high-quality (>90% competition and <5% contamination) and 72 to medium-quality (>50% completion and <10% contamination) MAGs (Table S6). The taxonomy was assigned using GTDB-tk and revealed 74 MAGs belonging to Bacteria and nine to Archaea (Fig. 3). Most of the bacterial MAGs corresponded to Proteobacteria, a few to the known NP-rich phylum Actinobacteria, and the remaining to Verrucomicrobria, Bacteroidetes, Nitrospinota, Marinisomatota and to the Deltaprotoebacteria clade SAR324 (Table S7). Bacteria from clade SAR324 (also referred to as marine group B) are abundant at different depths in the water column [79], but there are no reports of cultured isolates to date; this is also the case for Marinisomota and Nitrospinota. Arctic microbiomes appear to be enriched in members of Alpha- and Gammaproteobacteria, Actinobacteria, Bacteroidetes, Chlamydiae, and Parcubacteria [80]. The samples in this study were previously described [25] to be rich in Proteobacteria, and the phylotype ‘Candidatus Pelagibacter’ from the SAR11 clade identified as the most abundant one in epipelagic communities. Marinobacter and Alcanivorax, also had an abundant distribution across the NB and TR samples [25].

To understand the novelty of the recovered MAGs, ribosomal genes were extracted, and phylogenomic trees were also computed (Figs S5 and S6). Ribosomal genes were obtained from 12 of the 83 recovered MAGs. Identities to cultured strains present in NCBI nucleotide (nt) collection varied from 72–99%, highlighting the potential novelty of the recovered MAGs (Table S8). Phylogenomic trees, computed for MAGs from which representative genomes exist at the genus or order levels, revealed clades consisting only of MAGs recovered in this study. These were associated with the Verrucomicrobia phyla and Pelagibacteriales orders and likely correspond to uncharted genomic diversity (Figs S5 and S6).

To determine the BGC content in the recovered MAGs, we used antiSMASH analysis. In total, 179 BGCs were identified, represented by terpenes (*n*=77), other classes (45), NRPS (23), RiPPs (17), PKS (Type II/III) (13) and PKS Type I (three). From KnownClusterBlast analysis, a few of the BGCs exhibit high identity to the MIBiG entries for ectoine (Bin_43, Bin_46_1, Bin_128), β-carotein (Bin_56_1), carotenoid (Bin_84_10) and 4-formylaminooxyvinylglycine (Bin_142_1). The BGC data obtained for MAGs were generally consistent with those obtained for the assembled metagenome data (Table 3), with NRPS and lanthipeptides being the most abundant classes. Still, a higher number of NRPS and RiPPs BGCs was recovered from the MAGs.

The BGCs identified in MAGs were analysed in BiG-SCAPE, to determine their similarities to each other and also among the characterized MIBiG BGCs. Network analysis against the MIBiG database, computed with a 0.5 cutoff in BiG-SCAPE revealed that 136 out of the 179 BGCs analysed were singletons. At 1.0 distance cutoff it was possible to observe the existence of four singletons and 41 families (Fig. S7). Thus, the recovered BGCs seem to be diverse, belonging to different families.

**Fig. 4.**
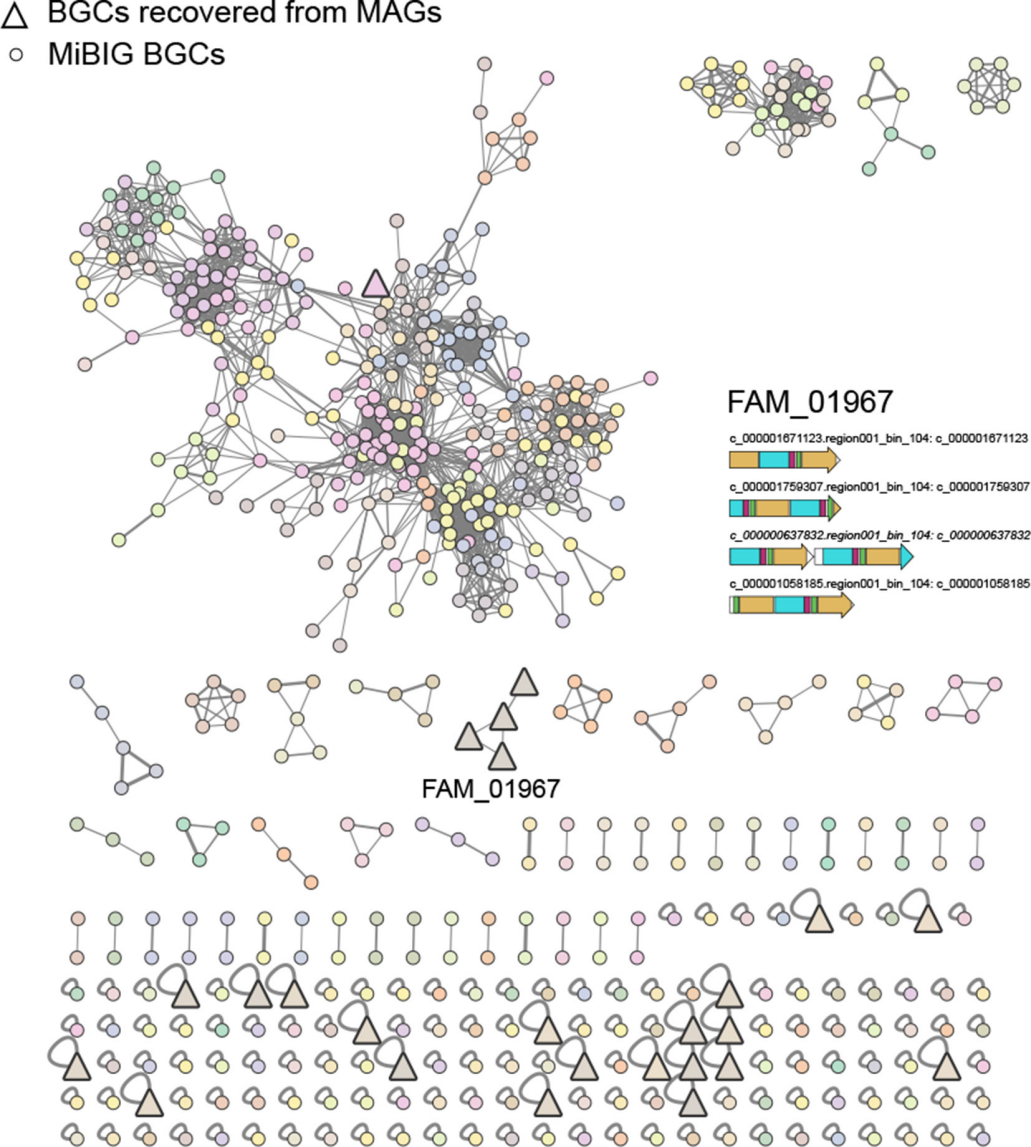
Network of NRPS BGCs recovered and MIBiG BGCs. BGCs recovered from the MAGs are represented by a bold triangle while MIBiG BGCs are represented by a circle. The different colours correspond to the different gene cluster families, identified by BiG-SCAPE. The BGC architecture of family FAM_01967 is represented.

**Fig. 5.**
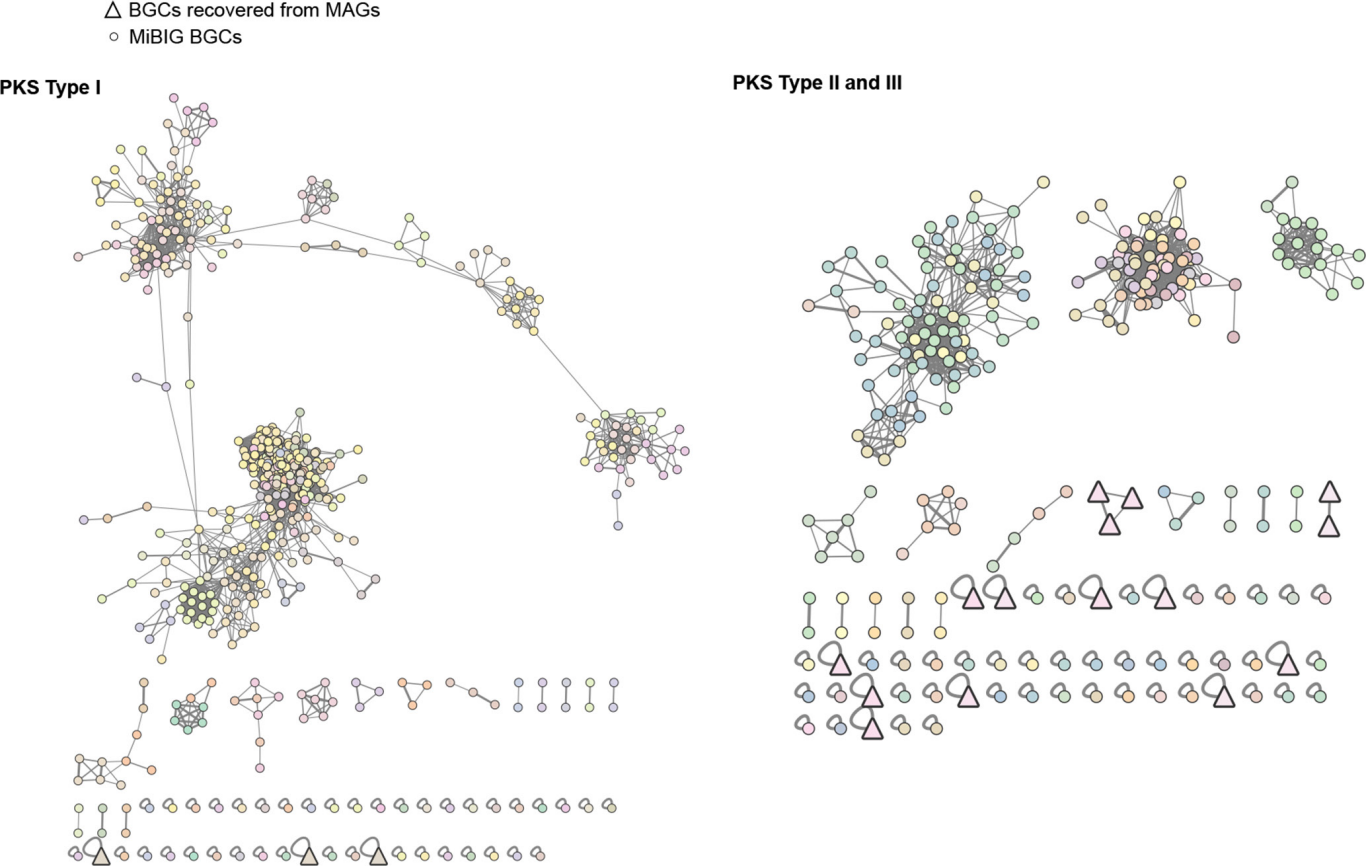
Network of PKS type I and PKS type II and III BGCs recovered and MIBiG BGCs. BGCs recovered from the MAGs are represented by a bold triangle while MIBiG BGCs are represented by a circle. The different colours correspond to the different gene cluster families, identified by BiG-SCAPE. The BGC architecture of family FAM_01967 is represented.

**Fig. 6.**
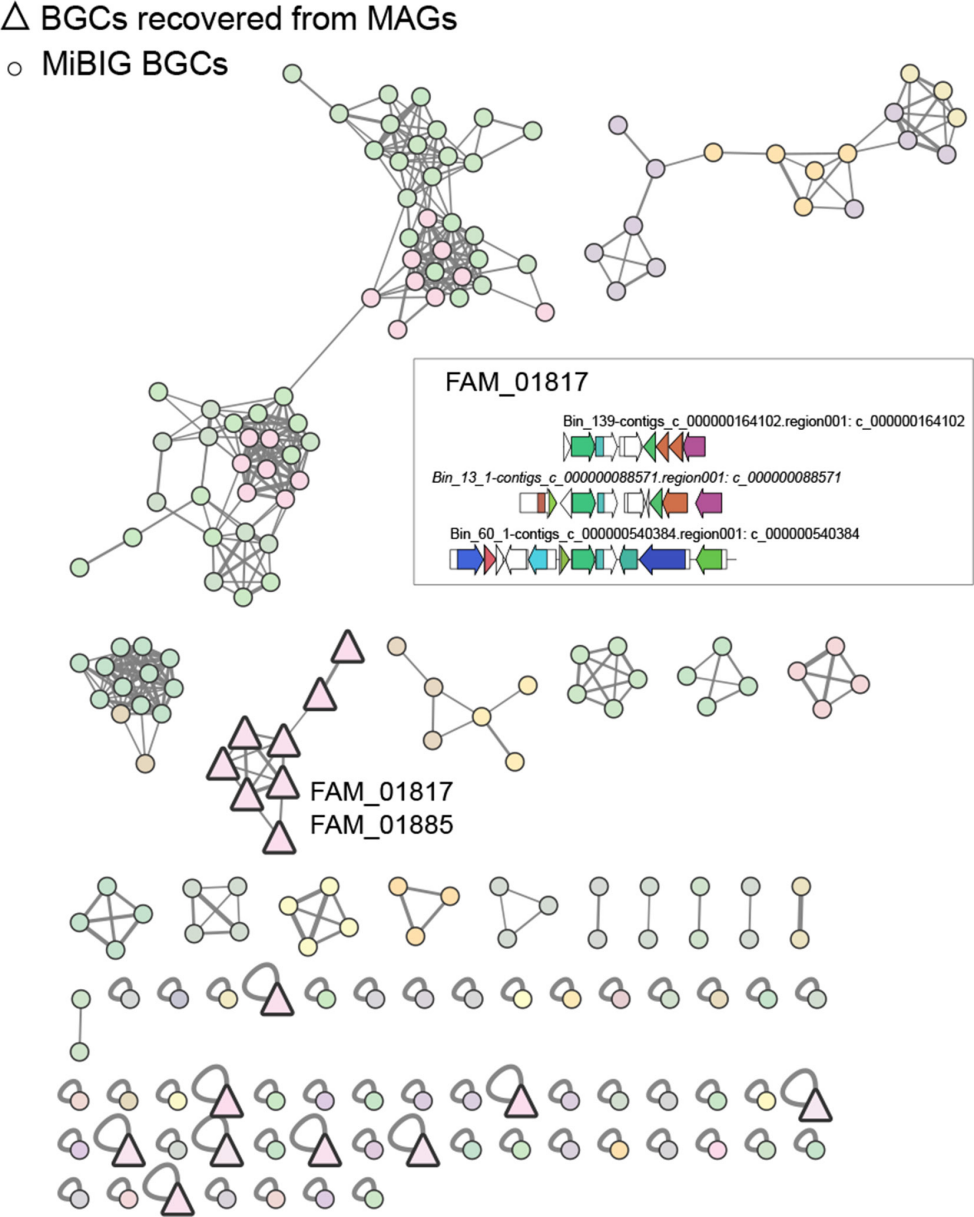
Network of RiPPs recovered and MIBiG BGCs. BGCs recovered from the MAGs are represented by a bold triangle while MIBiG BGCs are represented by a circle. The different colours correspond to the different gene cluster families, identified by BiG-SCAPE. The BGC architecture of family FAM_01817 is represented.

From the NRPS network (Fig. 4), and apart from a BGC that clustered with cyanopeptolin and anabaenopeptin BGCs (Fig. S8), the recovered BGCs either clustered in one family (FAM_01967) or were singletons, but were not associated with any MIBiG BGCs. BGCs from FAM_01967 belong to a high-quality MAG recovered from an actinobacterial *Rhodococcus sp*. At least one of the singleton BGCs seem to be complete (~44 kb) and shows 60% compositional identity to the indigoidine BGC [75] (Fig. S9). For the remaining BGCs, no matches were obtained through KnownClusterBlast (antiSMASH). Regarding type I PKSs, three clusters were retrieved, and did not group with other BGCs (Fig. 5). From these, only one seems to be complete (~18 kb) and corresponds to the same high-quality *Rhodococcus sp*. MAG mentioned above. In the type II and type III PKSs network (Fig. 5), the 15 analysed MAG-derived BGCs cluster in two families and as singletons. A few singleton BGCs seem to be complete or near-complete, were not matched by KnownClusterBlast and thus represent good candidates for heterologous expression. In total, 17 BGCs were identified as RiPPs, and the BiG-SCAPE analysis clustered these into two families or were found to be singletons (Fig. 6). One of the families (FAM_01817) was composed of three complete or near complete bacteriocin BGCs that were closest to BGCs from genomes of *Alcanivorax* (Fig. S11, S12) and *Methylophaga* (Fig. S13) species. The recovered RiPP BGCs are only distantly related to MIBiG-deposited metabolites since they group in distinct gene cluster families, and therefore are likely to represent novel diversity (Fig. 6).

### Comparison between BGCs recovered from amplicon, metagenomes and MAGs

In this study, different approaches were employed to characterize the biosynthetic diversity in Arctic microbial samples. An amplicon-based strategy allowed the recovery of a higher number of PKS-KS domain OTUs, in particular from samples that had identified as having low PKS abundance from metagenome data (Fig. S3). Blast analysis of the recovered KS domain sequences against MIBiG database have revealed that amplicon approach was able to recover sequences encoding for potential new metabolites, since 36% of the recovered sequences did not have a match to the MIBiG database contrasting to 7% of the sequences recovered from metagenomes. The best blast hits obtained were also distinct in the two approaches, in exclusion of two sequences with hight identity to nostophycin (Tables S3 and S9).

**Fig. 7.**
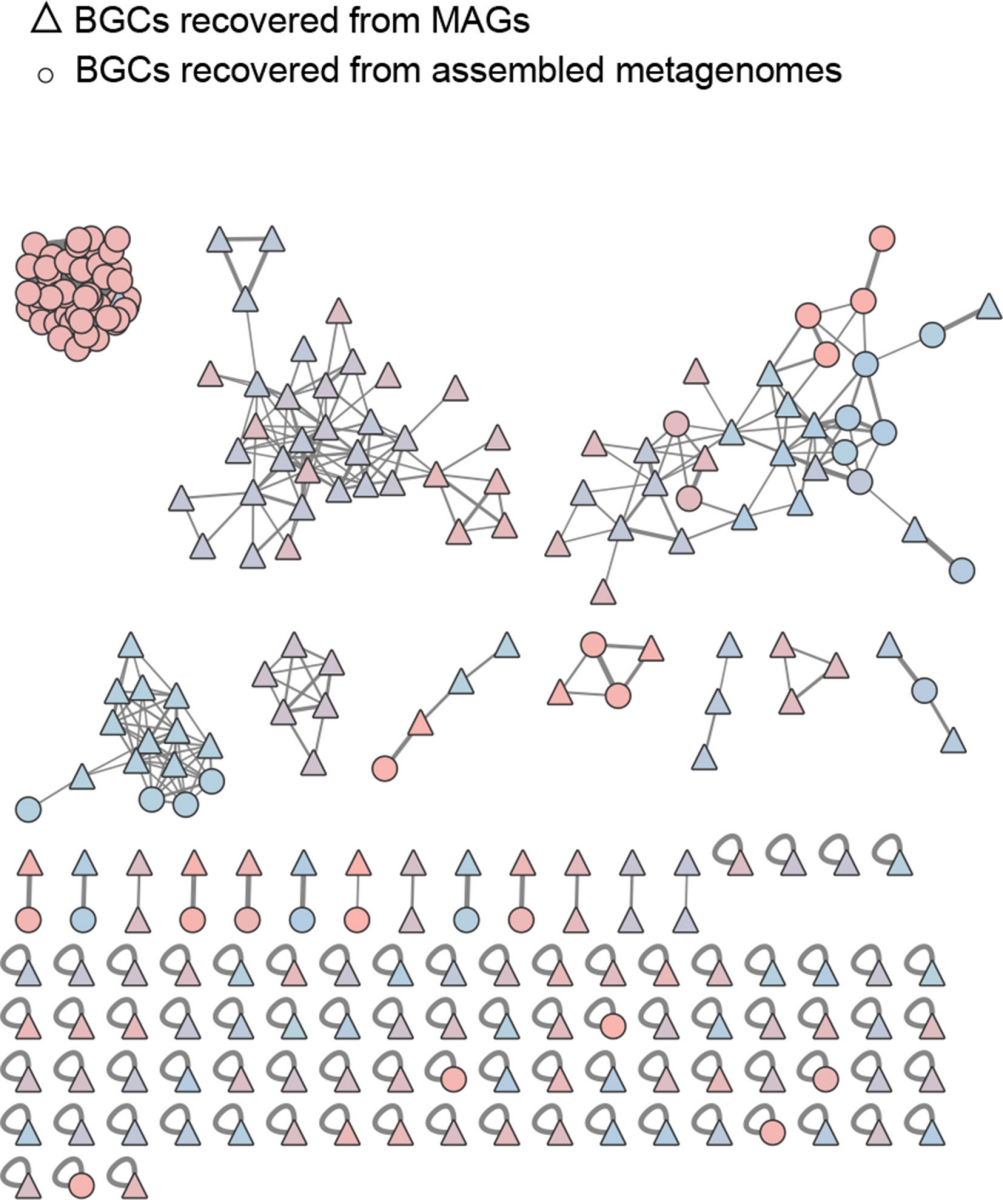
Network of BGCs recovered from the MAGs and from the metagenomes assembled using BiosyntheticSPades. MIBiG BGCs are not included. BGCs recovered from the MAGs are represented by a triangle while BGCs from metagenomes are represented by a circle. The different colours correspond to the different gene cluster families, identified by BiG-SCAPE.

We also employed two different assembly strategies for the shotgun metagenomics data, yielding assembled metagenomes or MAGs. To determine to which extent the recovered BGCs from these two approaches overlapped, a network using both sets of BGCs was computed in BiG-SCAPE (Fig. 7). We found several families composed exclusively of BGCs from MAGs, and a single family composed entirely of BGCs recovered from the assembled metagenome (biosyntheticSPAdes) in sample YP_20. Hence, at least for our samples, the assembly strategy is a major factor dictating BGC identification from shotgun metagenome data. The observed discrepancies are likely related to the completeness/fragmentation of the recovered BGCs, which influences the network construction in BiG-SCAPE. Also, for the MAGs reconstruction, we performed an initial step of co-assembly of data from the different, which might be responsible for the increase in the recovery of complete or near-complete genomes and consequently BGCs.

## Conclusions

We report here a microbial biosynthetic diversity assessment in Arctic Ocean samples. The integration of different methodological approaches (amplicon and metagenomics), as well as different assembly strategies, increased the number and the diversity of recovered BGCs. The amplicon-based strategy proved to be useful to specifically retrieve information regarding the KS domain even in samples with a low abundance of PKS gene. This strategy would be more appropriate to provide an overview of the biosynthetic diversity to select promising samples for further studies. Both metagenomic strategies enabled the recovery of complete or near-complete BGCs, including good candidates for heterologous expression. However, some BGCs were recovered exclusively by each of the methodologies, highlighting their complementarity. Sample YP_20 showed to be promising for further studies, as it harboured the highest number of OTUs without match to the MIBiG database and the highest number of clusters in antiSMASH. This particular sample corresponds to the only sample collected in early summer during an under-ice bloom of the haptophyte algae *Phaeocystis pouchetii* and this is likely the driving force behind a more diverse microbial community and a concomitantly richer biosynthetic repertoire.

The majority of the recovered BGCs in this study are distantly related to MIBiG BGCs, thus likely to be involved in the production of novel compounds, highlighting the potential of the Arctic Ocean for NPs discovery. Our approach can be a good starting point for bioprospection studies, since it directs future efforts towards biosynthetic rich samples and pinpoints candidate BGCs for heterologous expression.

## Supplementary Data

Supplementary material 1
