# Supplementary material for: Secondary metabolite biosynthetic diversity in Arctic Ocean metagenomes
Source: Microb Genom. 2021 Dec 14;7(12):000731. doi: 10.1099/mgen.0.000731 (PMC8767328; doi:10.1099/mgen.0.000731)
Supplement: Supplementary material 1 [file mgen-7-0731-s001.pdf]

Supplementary Information

Secondary metabolite biosynthetic diversity in Arctic Ocean metagenomes

Adriana Rego[1,2], Antonio Fernandez-Guerra[3], Pedro Duarte[4], Philipp Assmy[4], Pedro N. Leão[1*], Catarina Magalhães[1,5*]

[1] Interdisciplinary Centre of Marine and Environmental Research (CIIMAR), University of Porto, 4450-208 Matosinhos, Portugal
[2] Institute of Biomedical Sciences Abel Salazar (ICBAS), University of Porto, 4050-313 Porto, Portugal
[3]Microbial Genomics and Bioinformatics Research Group, Max Planck Institute for Marine Microbiology, Celsiusstraße 1, 28359, Bremen, Germany
[4] Norwegian Polar Institute, Fram Centre, N-9296 Tromsø, Norway
[5] Faculty of Sciences, University of Porto, 4150-179 Porto, Portugal

**corresponding authors: cmagalhaes@ciimar.up.pt and pleao@ciimar.up.pt*

Table S1 – Number of reads and sequences obtained during the pre-processing steps and number of predicted ORFs obtained through FragGenScan-Plus analysis.

		**Merging**		**Quality and Lenght trimming**						**Dereplication**	**Predicted ORFs**
**Sample**	Pairs	Merged	Not merged	Input (merged)	Qtrimmed	Result	Input (not-merged)	Qtrimmed	Result	Unique sequences	Number of predicted ORFs
**NB_5**	2569895	1512723 (58.9%)	1057172 (41.1%)	1512723	416372 (27.52%)	1509127 (99.76%)	2114344	2037248 (96.35%)	1814736 (85.83%)	3454605	3474762
**NB_50**	3045025	1728950 (56.8%)	1316075 (43.2%)	1728950	506326 (29.29%)	1726841 (99.88%)	2632150	2548593 (96.83%)	2257876 (85.78%)	4146285	4226932
**NB_250**	2208836	1106812 (50.1%)	1102024 (49.9%)	1106812	317981 (28.73%)	1105270 (99.86%)	2204048	2127030 (96.51%)	1922046 (87.21%)	3149891	3253686
**TR_50**	2322911	1233550 (53.1%)	1089361 (46.9%)	1233550	346532 (28.09%)	1231263 (99.81%)	2178722	2105909 (96.66%)	1864480 (85.58%)	3234281	3276752
**TR_250**	2027752	1000787 (49.4%)	1026965 (50.6%)	1000787	287891 (28.77%)	999639 (99.89%)	2053930	1986691 (96.73%)	1786924 (87.00%)	2902598	2975689
**YP_20**	3103984	1971681 (63.5%)	1132303 (36.5%)	1971681	399802 (20.28%)	1960172 (99.42%)	2264606	2134810 (94.27%)	306654 (13.54%)	4052967	4060707

Table S2– Distribution of PKS and NRPS domains in the samples under study.

		**Domain abundance**					
**BGC class**	**Domain**	**NB_5**	**NB_50**	**NB_250**	**TR_50**	**TR_250**	**YP_20**
**otherks**	hglD	38	36	4	15	11	8
**otherks**	hglE	46	38	13	23	39	16
**t1pks**	PKS_AT	436	456	394	403	317	673
**t1pks**	PKS_KS	110	102	24	57	79	140
**t2pks**	t2ks	2	2	1	2	7	2
**t3pks**	Chal_sti_synt_C	7	10	3	3	2	13
**t3pks**	Chal_sti_synt_N	6	9	3	7	0	5
**transatpks**	PKS_KS	110	102	24	57	79	140
**nrps**	AMP.binding	9468	11337	12316	11786	13424	17300
**nrps**	A.OX	310	357	259	300	431	619
**nrps**	Condensation	62	101	74	91	75	767

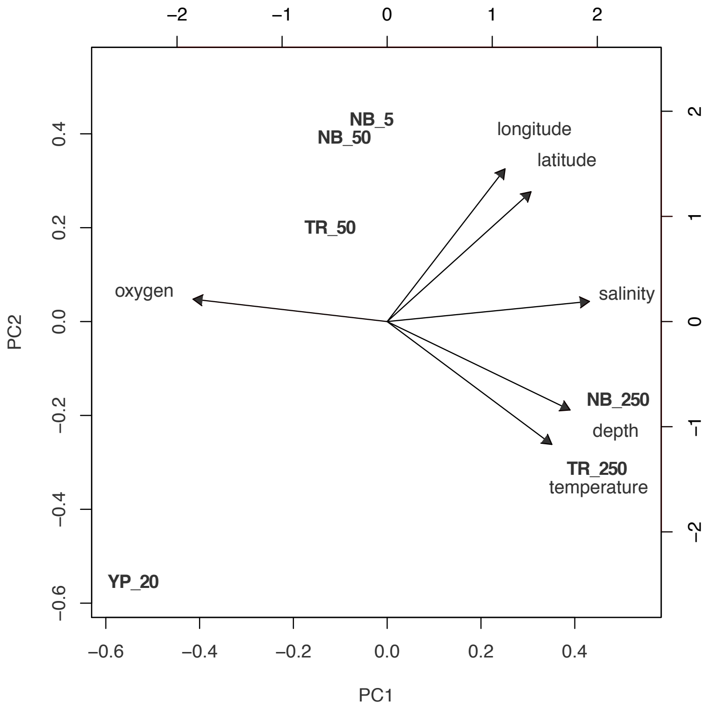

Figure S1 - Principal component analysis (PCA) applied to the environmental variables.

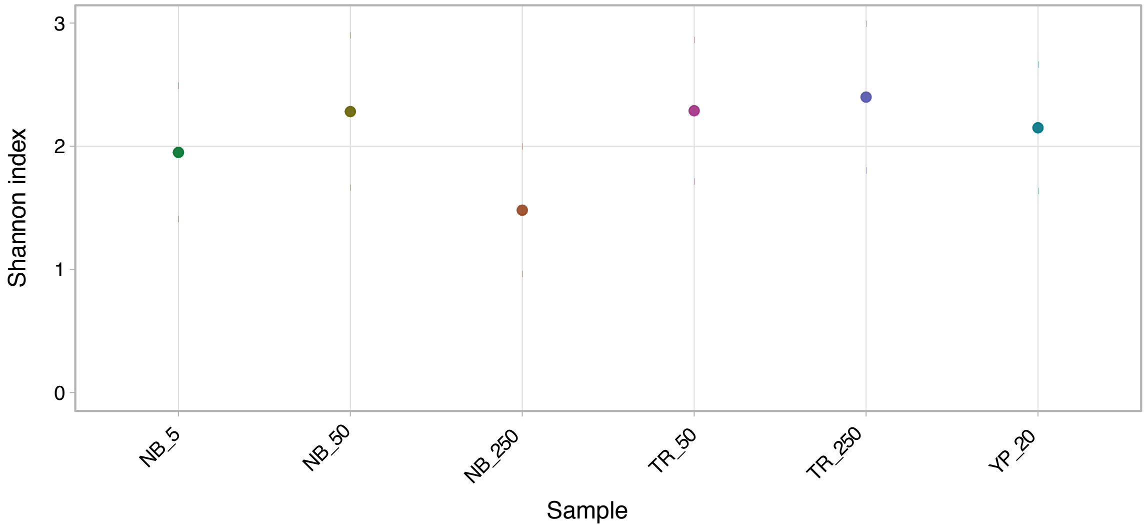

Figure S2 – Shannon diversity index of KS domains of PKS gene recovered from the metagenome obtained from BiG-Mex analysis.

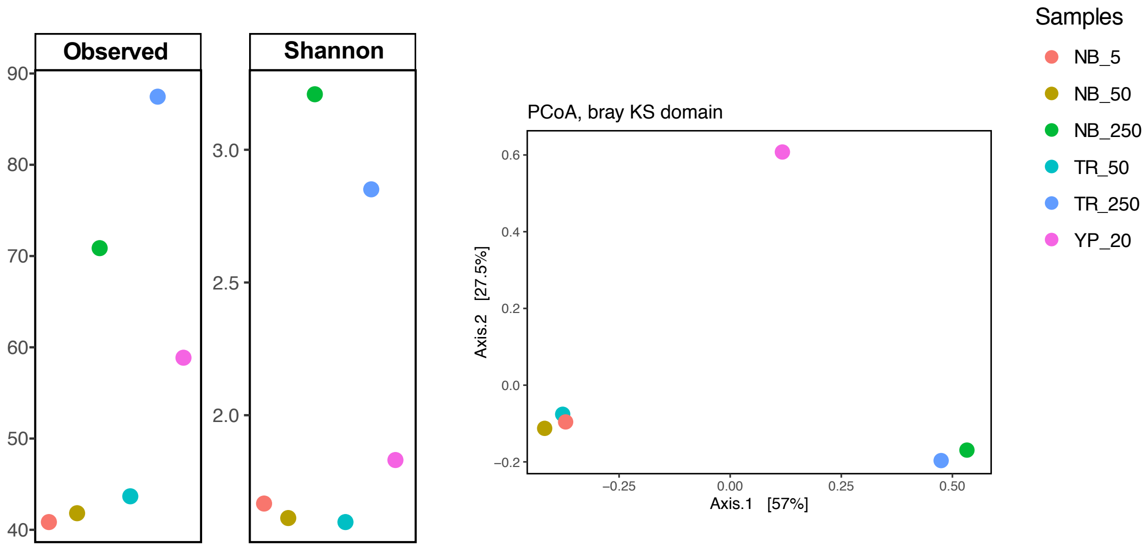

Figure S3 - Alpha and Beta-diversity plots of KS domain OTUs recovered. Alpha diversity metrics used were number of OTUs (grouped at 97% and in a second round at 95%) and Shannon diversity index.

Table S3 - Distribution of the 10 OTUs matching with higher identity to MiBIG database.

ID	main_product	Identity (%)	Query cover (%)	evalue	Bioactivity	Organism
**Uniq1404**	phenylnannolone A	89.130	92	$9.22e^{-20}$	multidrug resistance reversal agent	*Nannocystis pusilla* B150 (Deltaproteobacteria)
**Uniq581**	aculeximycin	78.947	96	$1.30e^{-22}$	antibiotic	*Streptosporangium albidum* (Actinobacteria)
**Uniq306**	carbamidocyclophane A - U	75.862	99	$1.11e^{-22}$	cytotoxic and antibiotic	*Nostoc* sp. CAVN 10 (Cyanobacteria)
**Uniq75**	jerangolid A/D	75.862	99	$1.17e^{-23}$	antifungal	*Sorangium cellulosum* (Deltaproteobacteria)
**Uniq1159**	BE-43547 A1-C2	75.610	99	$3.23e^{-35}$	cytotoxic and antibiotic	*Streptomyces* sp. (Actinobacteria)
**Uniq178**	nostophycin	73.333	99	$7.23e^{-22}$	weakly cytotoxic	*Nostoc* sp. 152 (Cyanobacteria)
**Uniq806**	carbamidocyclophane A - U	73.333	99	$1.59e^{-22}$	cytotoxic and antibiotic	*Nostoc* sp. CAVN 10 (Cyanobacteria)
**Uniq29**	nostophycin	72.414	99	$3.89e^{-22}$	weakly cytotoxic	*Nostoc* sp. 152 (Cyanobacteria)
**Uniq908**	mycobactin	72.152	98	$1.71e^{-35}$	siderophore	*Mycobacterium tuberculosis* (Actinobacteria)
**Uniq403**	phenalamide	71.667	99	$1.48e^{-21}$	anti-HIV	*Myxococcus stipitatus* DSM 14675 (Deltaproteobacteria)

Table S4– Quality filter of input samples. Number of sequences in adapter clipped files, quality filtered files and singletons.

	**Nr of sequences**
NB_5 clipped	2569895
NB_5 qc_clipped	2410222
NB_5 singletons	139094

NB_50 clipped	3045025
NB_50 qc_clipped	2849509
NB_50 singletons	171722
NB_250 clipped	2208836
NB_250 qc_clipped	2062992
NB_250 singletons	129742
TR_50 clipped	2322911
TR_50 qc_clipped	2295736
TR_50 singletons	144816
TR_250 clipped	2027752
TR_250 qc_clipped	2007454
TR_250 singletons	122409
YP_20 clipped	3103984
YP_20 qc_clipped	3062985
YP_20 singletons	149381

Table S5 –Blast result of recovered candidate BGCs against MiBIG database. The hits with >65% identity for each sample are represented.

Sample	Input	BGC_accession	Mibig_product	Identity (%)	Query cover (%)	evalue
**NB_5**	NODE_133_length_540_cluster_133_candidate_1	BGC0000866	polyhydroxyalkanoate	88.506	97	1.69e-110
**NB_5**	NODE_7_length_21236_cluster_7_candidate_1	BGC0000913	menaquinone	77.122	4	1.04e-142
**NB_50**	NODE_73_length_989_cluster_73_candidate_1	BGC0001155	GE2270	74.016	39	2.62e-63
**NB_50**	NODE_97_length_21381_cluster_97_candidate_1	BGC0000189	xenocyloins	68.579	5	1.53e-154
**NB_50**	NODE_85_length_713_cluster_85_candidate_1	BGC0000837	APE	65.816	82	1.31e-94
**NB_250**	NODE_65_length_785_cluster_64_candidate_1	BGC0000866	polyhydroxyalkanoate	85.99	79	3.05e-129
**NB_250**	NODE_24_length_52449_cluster_24_candidate_1	BGC0000781	O-antigen	76.777	2	0
**NB_250**	NODE_7_length_13240_cluster_7_candidate_1	BGC0001687	N-tetradecanoyl	71.238	12	0
**NB_250**	NODE_22_length_67483_cluster_22_candidate_1	BGC0001687	N-tetradecanoyl	71.023	2	0
**NB_250**	NODE_1_length_44570_cluster_1_candidate_1	BGC0000864	eicosapentaenoic	68.373	4	0
**NB_250**	NODE_16_length_51973_cluster_16_candidate_1	BGC0000791	O-antigen	68.035	2	4.57e-152
**NB_250**	NODE_81_length_16855_cluster_80_candidate_1	BGC0001120	burkholderic	65.217	9	0
**TR_50**	NODE_82_length_1661_cluster_83_candidate_1	BGC0000774	lipopolysaccharide	72.115	38	9.09e-92

**TR_50**	NODE_153_length_5918_cluster_154_candidate_1	BGC0000270	simocyclinone	68.525	15	3.26e-128
**TR_250**	NODE_186_length_5461_cluster_187_candidate_1	BGC0000270	simocyclinone	68.197	17	9.2e-130
**TR_250**	NODE_204_length_4066_cluster_205_candidate_1	BGC0000270	simocyclinone	68.197	22	5.68e-132
**TR_250**	NODE_106_length_1701_cluster_106_candidate_1	BGC0000864	eicosapentaenoic	66.972	19	4.26e-43
**YP_20**	NODE_202_length_42436_cluster_152_candidate_1	BGC0000700	istamycin	81.464	6	0
**YP_20**	NODE_18_length_1965_cluster_18_candidate_1	BGC0000838	flexirubin	71.585	56	0
**YP_20**	NODE_28_length_33037_cluster_28_candidate_1	BGC0001011	meridamycin	70.736	5	0
**YP_20**	NODE_210_length_10271_cluster_160_candidate_1	BGC0001687	N-tetradecanoyl	70.265	15	0
**YP_20**	NODE_56_length_17984_cluster_56_candidate_1	BGC0001411	polysaccharide	69.67	9	6.13e-154
**YP_20**	NODE_74_length_3614_cluster_74_candidate_1	BGC0001114	thailanstatin	69.521	33	1.71e-180
**YP_20**	NODE_50_length_27562_cluster_50_candidate_1	BGC0000270	simocyclinone	69.128	3	1.22e-114
**YP_20**	NODE_27_length_51220_cluster_27_candidate_1	BGC0000698	hygromycin	67.216	3	0
**YP_20**	NODE_49_length_37229_cluster_49_candidate_1	BGC0000038	coelimycin	66.78	4	0
**YP_20**	NODE_144_length_73002_cluster_144_candidate_1	BGC0000453	valinomycin	66.546	3	0

Table S6 – Completeness and redundancy of recovered MAGs, obtained using Anvi'o. High quality MAGs are identified in bold in Bin_ID.

**Bin_ID**	**Completeness (%)**	**Redundancy (%)**
**Bin_42**	100	4.23
**Bin_104**	98.59	4.23
**Bin_120**	98.59	0
**Bin_13_2**	98.59	2.82
**Bin_30**	97.18	2.82
**Bin_40**	95.77	1.41
**Bin_133_1**	95.77	2.82
**Bin_100_1**	95.77	1.41
**Bin_16_1**	95.77	4.23
**Bin_112_1**	91.55	2.82
**Bin_66_1**	90.79	3.95
**Bin_118**	87.32	2.82
**Bin_23_1**	87.32	2.82
**Bin_72**	85.92	4.23
**Bin_19**	84.51	2.82
**Bin_93**	84.21	1.32
**Bin_34**	83.1	0
**Bin_128**	81.69	4.23
**Bin_60_1**	80.28	7.04
**Bin_77_1**	80.28	4.23
**Bin_127**	75	3.95
**Bin_145_2**	74.65	5.63
**Bin_60_2**	74.65	1.41
**Bin_106**	74.65	8.45
**Bin_152**	73.68	6.58
**Bin_51**	73.24	4.23
**Bin_110**	73.24	5.63

**Bin_43**	73.24	2.82
**Bin_115**	71.83	8.45
**Bin_96**	70.42	9.86
**Bin_65**	69.01	7.04
**Bin_94_1**	67.61	4.23
**Bin_77_2**	67.61	5.63
**Bin_80**	67.61	8.45
**Bin_142_6_3**	66.2	7.04
**Bin_139**	64.79	8.45
**Bin_90_2**	64.79	4.23
**Bin_73**	63.38	4.23
**Bin_58_1**	63.38	9.86
**Bin_9_1**	63.38	9.86
**Bin_47**	63.38	7.04
**Bin_142_6_4**	63.38	7.04
**Bin_86_1**	63.16	9.21
**Bin_8_3**	61.97	9.86
**Bin_84_1_2**	61.97	9.86
**Bin_151_2**	60.56	9.86
**Bin_10_1**	60.56	4.23
**Bin_142_6_1**	60.56	7.04
**Bin_117_2**	60.53	3.95
**Bin_63_1_1**	6.02	3.61
**Bin_57**	59.15	7.04
**Bin_84_4_2**	59.15	7.04
**Bin_36_1**	57.75	9.86
**Bin_85_1**	57.75	8.45
**Bin_54_3**	57.75	8.45
**Bin_142_2**	57.75	7.04
**Bin_153_1**	57.75	5.63
**Bin_24_11**	57.75	7.04
**Bin_13_1**	56.34	0
**Bin_142_1**	56.34	7.04
**Bin_56_6**	56.34	9.86
**Bin_86_2**	55.26	9.21
**Bin_136**	55.26	7.89
**Bin_84_10**	54.93	7.04
**Bin_84_1_1_2**	54.93	8.45
**Bin_9_2_1**	54.93	7.04
**Bin_58_3**	53.52	9.86
**Bin_56_1**	53.52	7.04
**Bin_56_4**	53.52	9.86
**Bin_84_3_1**	53.52	8.45
**Bin_137_1**	52.11	9.86
**Bin_109_2**	52.11	8.45
**Bin_75_1**	52.11	5.63
**Bin_56_2**	52.11	8.45
**Bin_8_2_1**	52.11	9.86
**Bin_83**	51.32	5.26
**Bin_58_2**	50.7	7.04
**Bin_142_3**	50.7	9.86
**Bin_46_1**	50.7	9.86
**Bin_56_5**	50.7	8.45
**Bin_84_2_3**	50.7	9.86
**Bin_84_6_2**	50.7	8.45
**Bin_142_4**	50.7	8.45

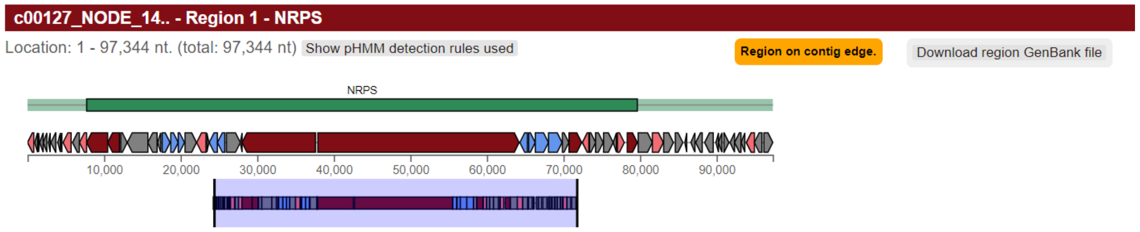

Figure S4 – NRPS BGC identified by antiSMASH with over 97 kb of length, from sample YP_20.

Table S7 – Taxonomy of each of the 83 medium and high-quality MAGs recovered. The taxonomy was assigned using GTDB-tk.

Bin_ID	Kingdom	Phylum	Class	Order	Family	Genus	Species
**Bin_10_1**	Bacteria	Proteobacteria	NA	NA	NA	NA	NA
**Bin_100_1**	Bacteria	Proteobacteria	Alphaproteobacteria	Rhodobacterales	Rhodobacteraceae	Sulfitobacter	Sulfitobacter pontiacus
**Bin_104**	Bacteria	Actinobacteriota	Actinobacteria	Mycobacteriales	Mycobacteriaceae	Rhodococcus	Rhodococcus sp000813105
**Bin_106**	Bacteria	Proteobacteria	Gammaproteobacteria	Pseudomonadales	Pseudohongiellaceae	UBA9145	NA
**Bin_109_2**	Bacteria	Proteobacteria	Gammaproteobacteria	Thiomicrospirales	Thioglobaceae	Thioglobus	NA
**Bin_110**	Bacteria	Verrucomicrobiota	Verrucomicrobiae	Pedosphaerales	UBA1100	NA	NA
**Bin_112_1**	Bacteria	SAR324	SAR324	SAR324	NAC60-12	Arctic96AD-7	NA
**Bin_115**	Bacteria	Proteobacteria	Alphaproteobacteria	UBA8366	GCA-2717185	NA	NA
**Bin_118**	Bacteria	Proteobacteria	Gammaproteobacteria	Pseudomonadales	Oleiphilaceae	Marinobacter	Marinobacter salarius
**Bin_120**	Bacteria	Proteobacteria	Gammaproteobacteria	Enterobacterales	Alteromonadaceae	Glaciecola	NA
**Bin_128**	Bacteria	Proteobacteria	Gammaproteobacteria	Pseudomonadales	Spongiibacteraceae	Zhongshania	Zhongshania aliphaticivorans
**Bin_13_1**	Bacteria	Proteobacteria	Gammaproteobacteria	Pseudomonadales	Alcanivoracaceae	Alcanivorax	NA
**Bin_13_2**	Bacteria	Proteobacteria	Alphaproteobacteria	Caulobacterales	Hyphomonadaceae	Hyphomonas	Hyphomonas sp000682775
**Bin_133_1**	Bacteria	Proteobacteria	Gammaproteobacteria	Pseudomonadales	Porticoccaceae	HTCC2207	NA
**Bin_137_1**	Bacteria	Proteobacteria	Gammaproteobacteria	AqS2	NA	NA	NA
**Bin_139**	Bacteria	Proteobacteria	Gammaproteobacteria	Pseudomonadales	Alcanivoracaceae	Alcanivorax	NA
**Bin_142_1**	Bacteria	Marinisomatota	Marinisomatia	Marinisomatales	Marinisomataceae	Marinisoma	NA
**Bin_142_2**	Bacteria	Proteobacteria	Alphaproteobacteria	Rhodobacterales	Rhodobacteraceae	GCA-002712045	NA
**Bin_142_3**	Bacteria	Proteobacteria	Alphaproteobacteria	Pelagibacterales	NA	NA	NA
**Bin_142_4**	Bacteria	Marinisomatota	Marinisomatia	Marinisomatales	TCS55	NA	NA
**Bin_142_6_1**	Bacteria	Proteobacteria	Gammaproteobacteria	SAR86	NA	NA	NA
**Bin_142_6_3**	Bacteria	Bacteroidota	Bacteroidia	Flavobacteriales	Flavobacteriaceae	MS024-2A	NA

**Bin_142_6_4**	Bacteria	Proteobacteria	Gammaproteobacteria	SAR86	NA	NA	NA
**Bin_145_2**	Bacteria	Verrucomicrobiota	Verrucomicrobiae	Verrucomicrobiales	Akkermansiaceae	UBA1315	NA
**Bin_151_2**	Bacteria	Proteobacteria	Gammaproteobacteria	UBA10353	LS-SOB	UBA11791	NA
**Bin_153_1**	Bacteria	Proteobacteria	Alphaproteobacteria	Pelagibacterales	Pelagibacteraceae	NA	NA
**Bin_16_1**	Bacteria	Proteobacteria	Alphaproteobacteria	Sphingomonadales	Sphingomonadaceae	Sphingopyxis_A	Sphingopyxis_A baekryungensis
**Bin_19**	Bacteria	Proteobacteria	Gammaproteobacteria	Pseudomonadales	Nitrincolaceae	ASP10-02a	ASP10-02a sp002335115
**Bin_23_1**	Bacteria	Nitrospinota	Nitrospinia	Nitrospinales	Nitrospinaceae	SCGCAAA288-L16	NA
**Bin_24_11**	Bacteria	Proteobacteria	Alphaproteobacteria	Pelagibacterales	Pelagibacteraceae	Pelagibacter	NA
**Bin_30**	Bacteria	Proteobacteria	Gammaproteobacteria	Burkholderiales	Burkholderiaceae	Ralstonia	Ralstonia pickettii
**Bin_34**	Bacteria	Marinisomatota	Marinisomatia	Marinisomatales	UBA1611	GCA-2722105	NA
**Bin_36_1**	Bacteria	Proteobacteria	Gammaproteobacteria	UBA10353	LS-SOB	REDSEA-S09-B13	NA
**Bin_40**	Bacteria	Marinisomatota	Marinisomatia	Marinisomatales	TCS55	TCS55	NA
**Bin_42**	Bacteria	Actinobacteriota	Acidimicrobiia	Microtrichales	MedAcidi-G1	UBA9410	NA
**Bin_43**	Bacteria	Proteobacteria	Gammaproteobacteria	Pseudomonadales	Porticoccaceae	Porticoccus	NA
**Bin_46_1**	Bacteria	Proteobacteria	Gammaproteobacteria	Pseudomonadales	Alcanivoraceae	NA	NA
**Bin_47**	Bacteria	Proteobacteria	Alphaproteobacteria	IN39	NA	NA	NA
**Bin_51**	Bacteria	Proteobacteria	Gammaproteobacteria	Pseudomonadales	HTCC2089	UBA9659	NA
**Bin_54_3**	Bacteria	Proteobacteria	NA	NA	NA	NA	NA
**Bin_56_1**	Bacteria	Proteobacteria	Alphaproteobacteria	Rhodobacterales	Rhodobacteraceae	Planktomarina	NA
**Bin_56_2**	Bacteria	Proteobacteria	NA	NA	NA	NA	NA
**Bin_56_4**	Bacteria	Proteobacteria	Alphaproteobacteria				NA
**Bin_56_5**	Bacteria	Proteobacteria	NA	NA	NA	NA	NA
**Bin_56_6**	Bacteria	Proteobacteria	NA	NA	NA	NA	NA
**Bin_57**	Bacteria	Actinobacteriota	Acidimicrobiia	Microtrichales	TK06	UBA9040	NA
**Bin_58_1**	Bacteria	Proteobacteria	Alphaproteobacteria	Pelagibacterales	Pelagibacteraceae	NA	NA
**Bin_58_2**	Bacteria	Proteobacteria	Alphaproteobacteria	Pelagibacterales	Pelagibacteraceae	NA	NA
**Bin_58_3**	Bacteria	Proteobacteria	Alphaproteobacteria	Pelagibacterales	Pelagibacteraceae	Pelagibacter	NA
**Bin_60_1**	Bacteria	Proteobacteria	Gammaproteobacteria	Nitrosococcales	Methylophagaceae	NA	NA
**Bin_60_2**	Bacteria	Proteobacteria	Gammaproteobacteria	Pseudomonadales	Porticoccaceae	HTCC2207	NA
**Bin_63_1_1**	Bacteria	Proteobacteria	Alphaproteobacteria	HIMB59	NA	NA	NA
**Bin_65**	Bacteria	Verrucomicrobiota	Verrucomicrobiae	Pedosphaerales	UBA1096	UBA1096	NA
**Bin_72**	Bacteria	Proteobacteria	Alphaproteobacteria	Sphingomonadales	Sphingomonadaceae	Erythrobacter_A	NA
**Bin_73**	Bacteria	Proteobacteria	Gammaproteobacteria	Pseudomonadales	Moraxellaceae	Psychrobacter	NA

**Bin_75_1**	Bacteria	Proteobacteria	Gammaproteobacteria	Pseudomonadales	Alcanivoracaceae	Alcanivorax	NA
**Bin_77_1**	Bacteria	Proteobacteria	Gammaproteobacteria	UBA11654	UBA11654	NA	NA
**Bin_77_2**	Bacteria	Bacteroidota	Bacteroidia	Flavobacteriales	Flavobacteriaceae	MED-G11	MED-G11 sp002729755
**Bin_8_2_1**	Bacteria	Bacteroidota	Bacteroidia	Flavobacteriales	BACL11	NA	NA
**Bin_8_3**	Bacteria	Proteobacteria	Gammaproteobacteria	NA	NA	NA	NA
**Bin_80**	Bacteria	Actinobacteriota	Acidimicrobiia	Microtrichales	MedAcidi-G1	NA	NA
**Bin_84_1_1_2**	Bacteria	Proteobacteria	Alphaproteobacteria	Pelagibacterales	Pelagibacteraceae	NA	NA
**Bin_84_1_2**	Bacteria	Proteobacteria	Alphaproteobacteria	Pelagibacterales	Pelagibacteraceae	NA	NA
**Bin_84_10**	Bacteria	Proteobacteria	Alphaproteobacteria	Pelagibacterales	NA	NA	NA
**Bin_84_2_3**	Bacteria	Proteobacteria	Alphaproteobacteria	Pelagibacterales	Pelagibacteraceae	NA	NA
**Bin_84_3_1**	Bacteria	Proteobacteria	Alphaproteobacteria	Pelagibacterales	Pelagibacteraceae	NA	NA
**Bin_84_4_2**	Bacteria	Proteobacteria	Alphaproteobacteria	Pelagibacterales	Pelagibacteraceae	NA	NA
**Bin_84_6_2**	Bacteria	Proteobacteria	Alphaproteobacteria	Pelagibacterales	NA	NA	NA
**Bin_85_1**	Bacteria	Proteobacteria	Gammaproteobacteria	Thiomicrospirales	Thioglobaceae		NA
**Bin_9_1**	Bacteria	UBP7	NA	NA	NA	NA	NA
**Bin_9_2_1**	Bacteria	Proteobacteria	Alphaproteobacteria	NA	NA	NA	NA
**Bin_90_2**	Bacteria	Proteobacteria	Gammaproteobacteria	Pseudomonadales	Oleiphilaceae	Marinobacter	NA
**Bin_94_1**	Bacteria	Proteobacteria	Gammaproteobacteria	Thiomicrospirales	Thioglobaceae		NA
**Bin_96**	Bacteria	Verrucomicrobiota	Verrucomicrobiae	Verrucomicrobiales	Akkermansiaceae	SW10	NA
**Bin_83**	Archaea	Thermoplasmatota	Poseidoniia	Poseidoniales	Thalassoarchaeaceae	MGIIb-O1	MGIIb-O1 sp002498525
**Bin_127**	Archaea	Thermoplasmatota	Poseidoniia	Poseidoniales	Thalassoarchaeaceae	Thalassarchaeum	Thalassarchaeum sp002495735
**Bin_66_1**	Archaea	Thermoplasmatota	Poseidoniia	Poseidoniales	Thalassoarchaeaceae	MGIIb-O2	NA
**Bin_86_1**	Archaea	Thermoplasmatota	Poseidoniia	Poseidoniales	Poseidoniaceae	MGIIa-L1	NA
**Bin_86_2**	Archaea	Thermoplasmatota	Poseidoniia	Poseidoniales	Poseidoniaceae	MGIIa-L1	NA
**Bin_93**	Archaea	Thermoplasmatota	Poseidoniia	MGIII	CG-Epi1	CG-Epi1	NA
**Bin_117_2**	Archaea	Crenarchaeota	Nitrososphaeria	Nitrososphaerales	Nitrosopumilaceae	Nitrosopumilus	NA
**Bin_136**	Archaea	Crenarchaeota	Nitrososphaeria	Nitrososphaerales	Nitrosopumilaceae	Nitrosopumilus	NA
**Bin_152**	Archaea	Crenarchaeota	Nitrososphaeria	Nitrososphaerales	Nitrosopumilaceae	Nitrosopelagicus	NA

Table S8 – Blast(n) of ribosomal genes recovered from the MAGs against nucleotide collection (nt) with uncultured excluded.

Bin_ID	Completeness (%)	Redundancy (%)	Identity (%)	Query cover (%)	Accession number	Description
**Bin_23_1 (1) 23S – uncultured excluded**	87.32	2.82	86.39	99	AP022810.1	Desulfuromonas sp. AOP6 DNA, complete genome
**Bin_23_1 (2) 23S – uncultured excluded**	87.32	2.82	76.06	99	CP010070.1	Candidatus Methanoplasma termitum strain MpT1, complete genome
**Bin_60_1 – 16S – uncultured excluded**	80.28	7.04	94.47	99	AM402960.1	bacterial endosymbiont of Idas sp. partial 16S rRNA gene, clone M2.41
**Bin_77_1 – uncultured excluded**	80.28	4.23	89.29	100	CP016268.1	Woeseia oceani strain XK5, complete genome
**Bin_96 – 16S uncultured excluded**	70.42	9.86	91.10	76	HQ675573.1	Verrucomicrobia bacterium SCGC AAA240-E08 small subunit ribosomal RNA gene, partial sequence
**Bin_151_2 - 16S – uncultured excluded**	60.56	9.86	83.65	100	CP042829.1	Tepidiforma bonchosmolovskayae strain 3753O chromosome, complete genome
**Bin_57-16S (uncultured excluded)**	59.15	7.04	88.38	99	KP174508.1	Bacterium YC-ZSS-LKJ199 16S ribosomal RNA gene, partial sequence
**Bin_36_1 16S – uncultured excluded**	57.75	9.86	88.18	97	NR_151905.1	Vicinamibacter silvestris strain Ac_5_C6 16S ribosomal RNA
**Bin_54_3 - 23S uncultured excluded**	57.75	8.45	72.98	100	NR_076762.1	Dehalogenimonas lykanthroporepellens strain BL-DC-9 23S ribosomal RNA gene, complete sequence
**Bin_142_1 - 16S uncultured excluded**	56.34	7.04	83	97	AY344400.1	Unidentified bacterium clone K2-30-7 16S ribosomal RNA gene, partial sequence
**Bin_142_1 – 23 S uncultured excluded**	56.34	7.04	76.21	99	CP000859.1	Desulfococcus oleovorans Hxd3, complete genome
**Bin_137_1 (1) Uncultured excluded**	52.11	9.86	90.54	99	FM202063.1	Inanidrilus exumae Alpha 1a endosymbiont 16S rRNA gene, clone Iexu2_29_7
**Bin_137_1 (2) -28S – uncultured excluded**	52.11	9.86	79.14	100	DQ779991.1	Gymnodinium aureolum strain GrAr01 18S ribosomal RNA gene, partial sequence
**Bin_137_1 (3) -23S uncultured excluded**	52.11	9.86	78.21	99	CP036280.1	Planctomycetes bacterium Pan265 chromosome, complete genome

**Bin_56_2 (1) – 16S – uncultured excluded**	52.11	8.45	98.92	100	KC900889.1	Phaeocystis globosa strain Pg-G(A) chloroplast, complete genome
**Bin_56_2 (2) – 28S uncultured excluded**	52.11	8.45	98.83	98	AF289040.1	Phaeocystis antarctica internal transcribed spacer 2, partial sequence; and 28S ribosomal RNA gene, complete sequence
**Bin_56_5 - 16S - uncultured excluded**	50.7	8.45	98.24	100	FJ858267.1	Micromonas sp. RCC299 chloroplast, complete genome
**Bin_56_5 - 23S nt uncultured excluded**	50.7	8.45	99.81	100	FO082258.2	Bathycoccus prasinos genomic : Mitochondrion

Table S9– Result from KnownCluster Blast for Bin_43 with >65% identity.

ctg953_2	BGC0000859|c1|2203-2607|+|ACB30332.1|ectoine_synthase|ACB30332.1	87.2	1.0e-67
**ctg953_2**	BGC0000855|c1|2269-2673|+|ABB30172.1|ectoine_synthase|ABB30172.1	87.1	1.2e-66
**ctg953_3**	BGC0000857|c1|1709-3037|+|ABY40745.1|L-2,4-diaminobutyrate_aminotransferase|ABY40745.1	87.0	1.3e-66
**ctg953_2**	BGC0000854|c1|3652-4056|+|AAY96772.1|ectoine_synthase|AAY96772.1	86.5	2.3e-67
**ctg953_2**	BGC0000857|c1|3083-3487|+|ABY40746.1|ectoine_synthase|ABY40746.1	85.8	6.8e-67
**ctg953_3**	BGC0000859|c1|820-2151|+|ACB30331.1|L-2,4-diaminobutyric_acid_aminotransferase|ACB30331.1	83.8	3.9e-63
**ctg953_3**	BGC0000854|c1|2270-3601|+|AAY96771.1|L-2,4-diaminobutyrat_transaminase|AAY96771.1	83.8	5.0e-63
**ctg953_3**	BGC0000856|c1|1088-2425|+|ABY40740.1|L-2,4-diaminobutyrate_aminotransferase|ABY40740.1	82.4	3.9e-63
**ctg953_2**	BGC0000856|c1|2497-2901|+|ABY40741.1|ectoine_synthase|ABY40741.1	79.5	1.7e-62
**ctg953_1**	BGC0000856|c1|3034-4347|+|ABY40742.1|aspartokinase|ABY40742.1	73.9	9.1e-137
**ctg953_1**	BGC0000857|c1|3558-4871|+|ABY40747.1|aspartokinase|ABY40747.1	73.6	8.6e-135
**ctg953_1**	BGC0000854|c1|4117-5559|+|AAY96773.1|aspartokinase|AAY96773.1	72.6	4.7e-133
**ctg953_3**	BGC0000855|c1|788-2098|+|ABB30171.1|L-2,4-diaminobutyric_acid_acetyl_transferase|ABB30171.1	67.9	4.4e-51
**ctg953_1**	BGC0000859|c1|2667-3305|+|ACB30333.1|aspartokinase|ACB30333.1	66.7	3.2e-81

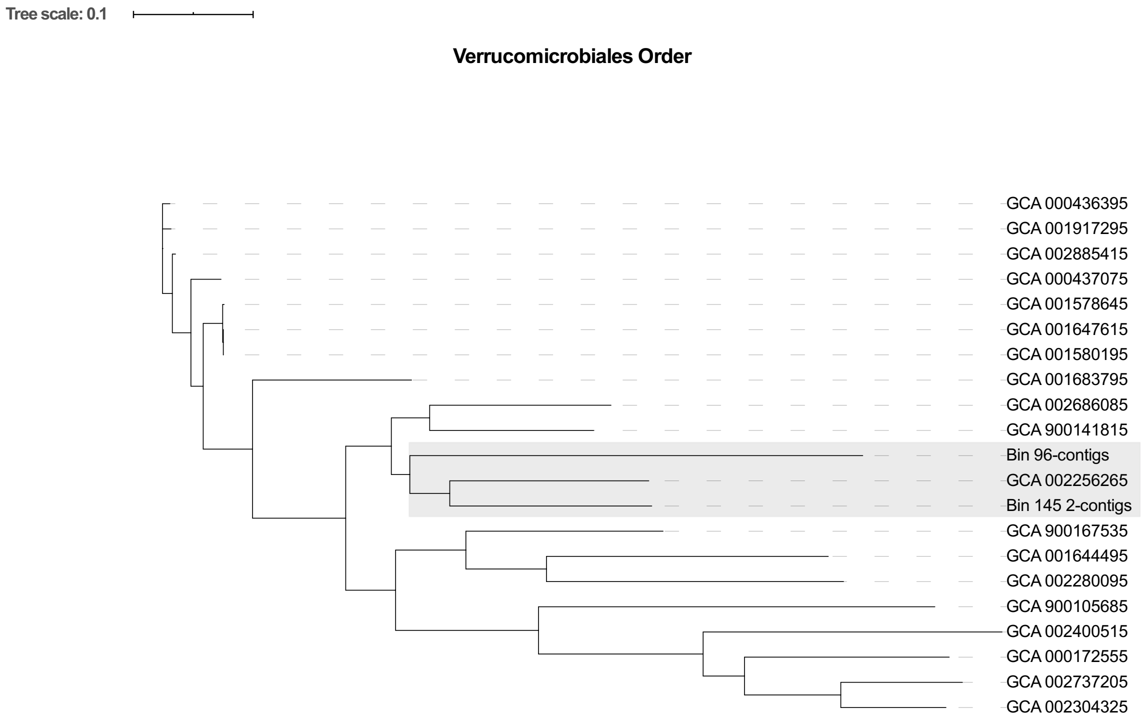

Figure S5 – Phylogenomic tree of Verrucomicrobiales order computed using PhyloPhlan. In the phylogenomic tree a clade harbouring only recovered MAGs is highlighted.

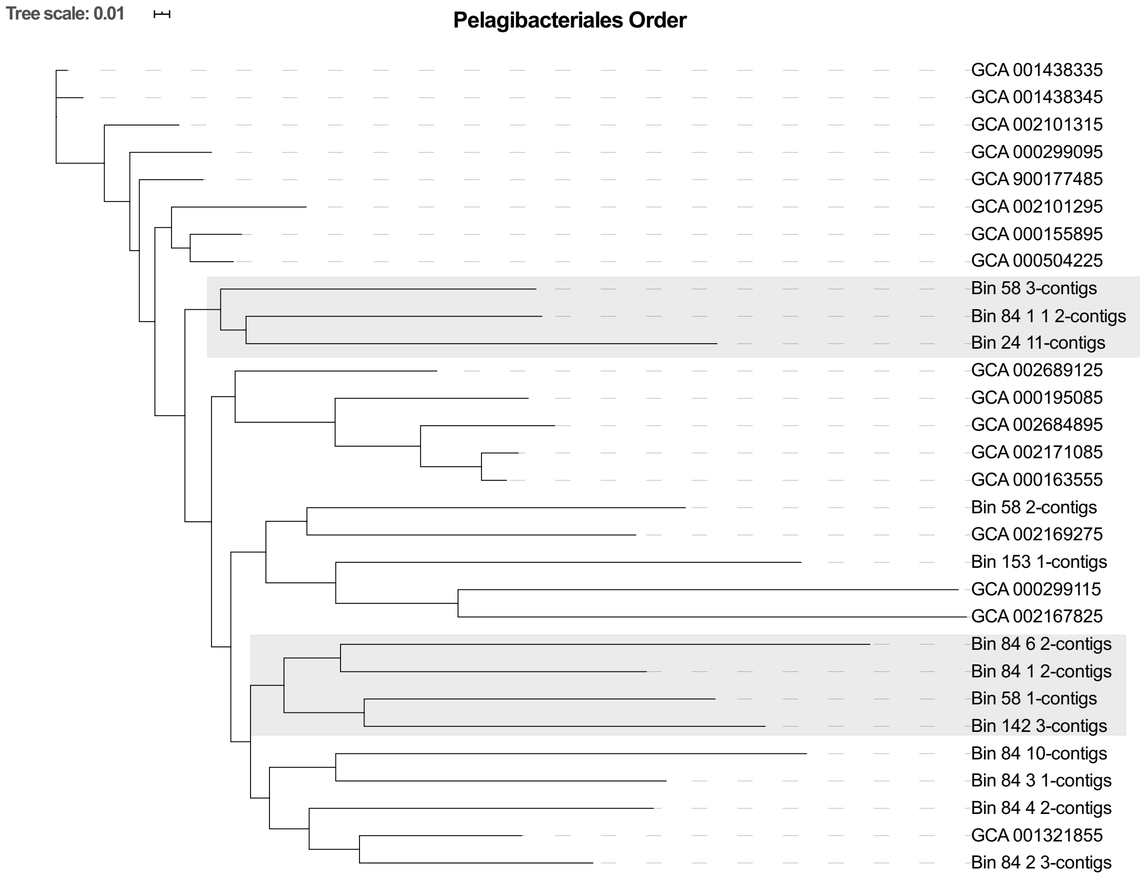

Figure S6– Phylogenomic tree of Pelagibacteriales order computed using PhyloPhlan. In the phylogenomic tree clades harbouring only recovered MAGs are highlighted.

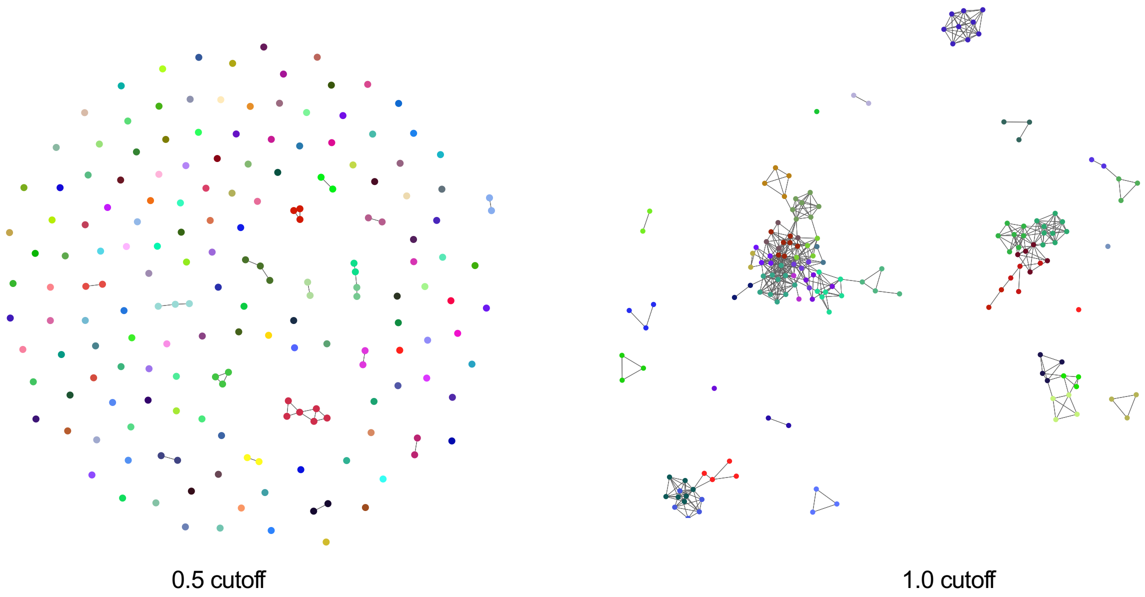

Figure S7 - Network of recoverd BGCs, computed with a 0.5 and a 1.0 cutoff in BiG-SCAPE. From the 178 BGCs imported, at 0.5 cutoff 136 are singletons. At 1.0 cutoff it is possible to observe the existence of 4 singletons and 41 families.

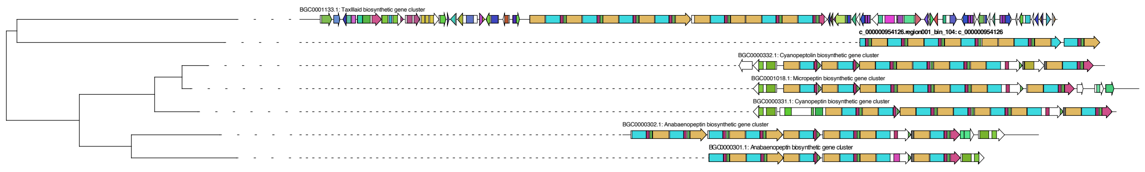

Figure S8 – NRPS BGCs from family FAM_01002 including one MAG recovered BGC and MiBiG BGCs.

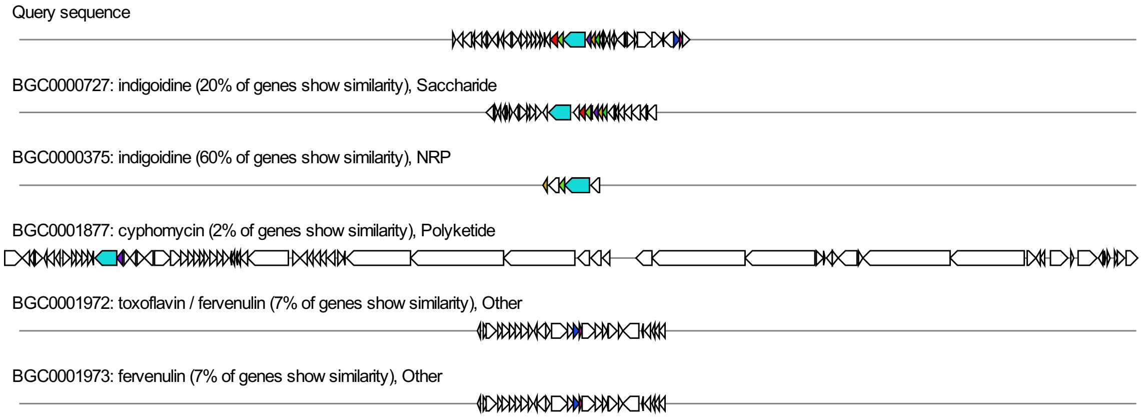

Figure S9 – Knowncluster Blast for NRPS c_000000759566 from MAG Bin_104.

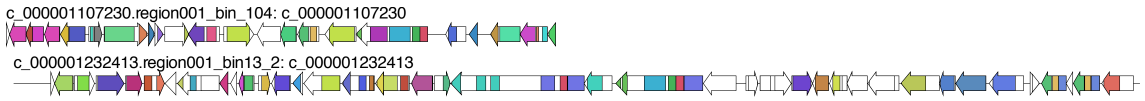

Figure S10 – Complete or near-complete singleton BGCs of PKS Type II and III network.

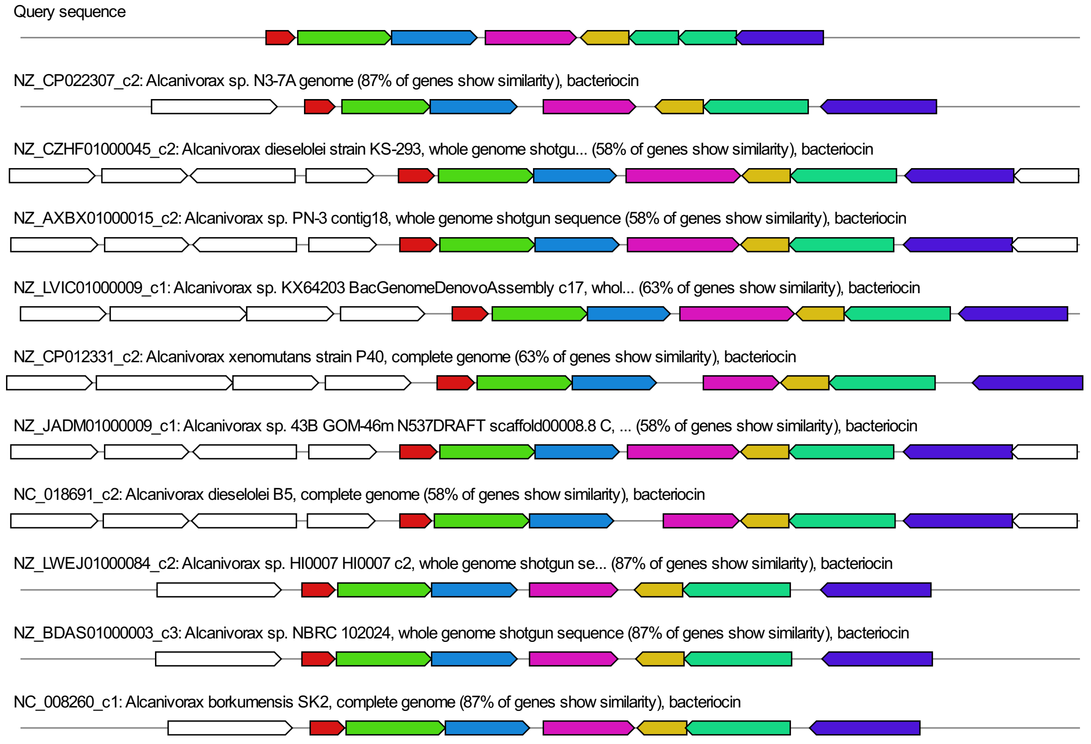

Figure S11 - AntiSMASH clusterblast output from a bacteriocin recovered from MAG Bin_139.

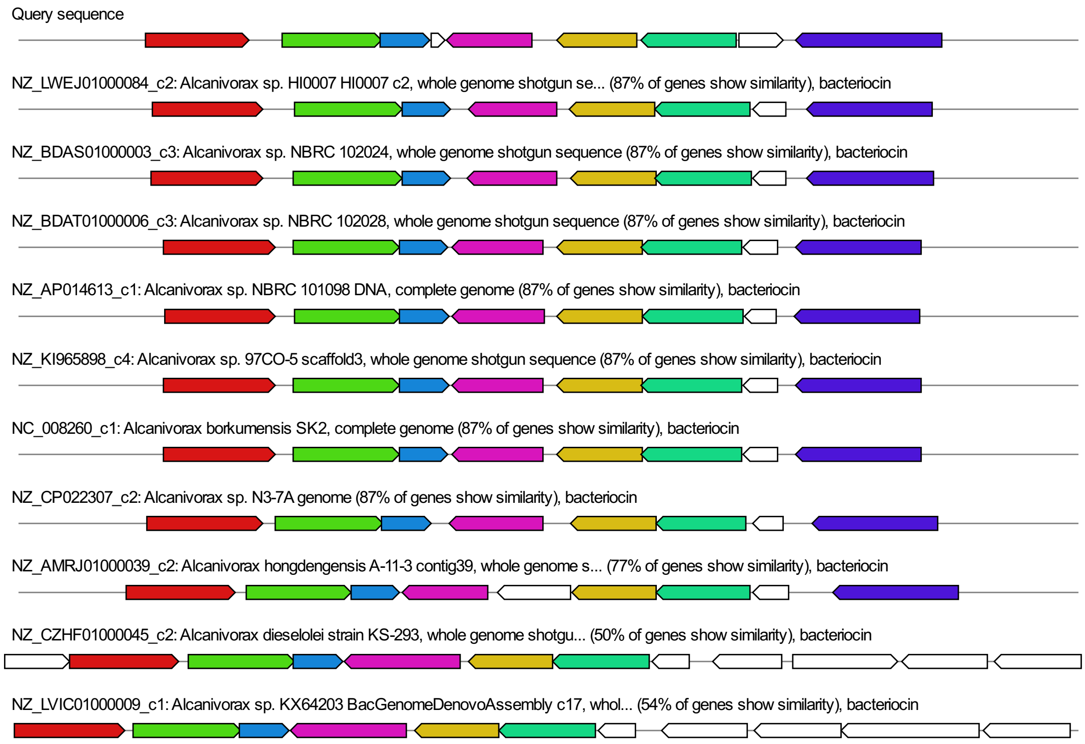

Figure S12 – AntiSMASH clusterblast output from a bacteriocin recovered from MAG Bin_13_1.

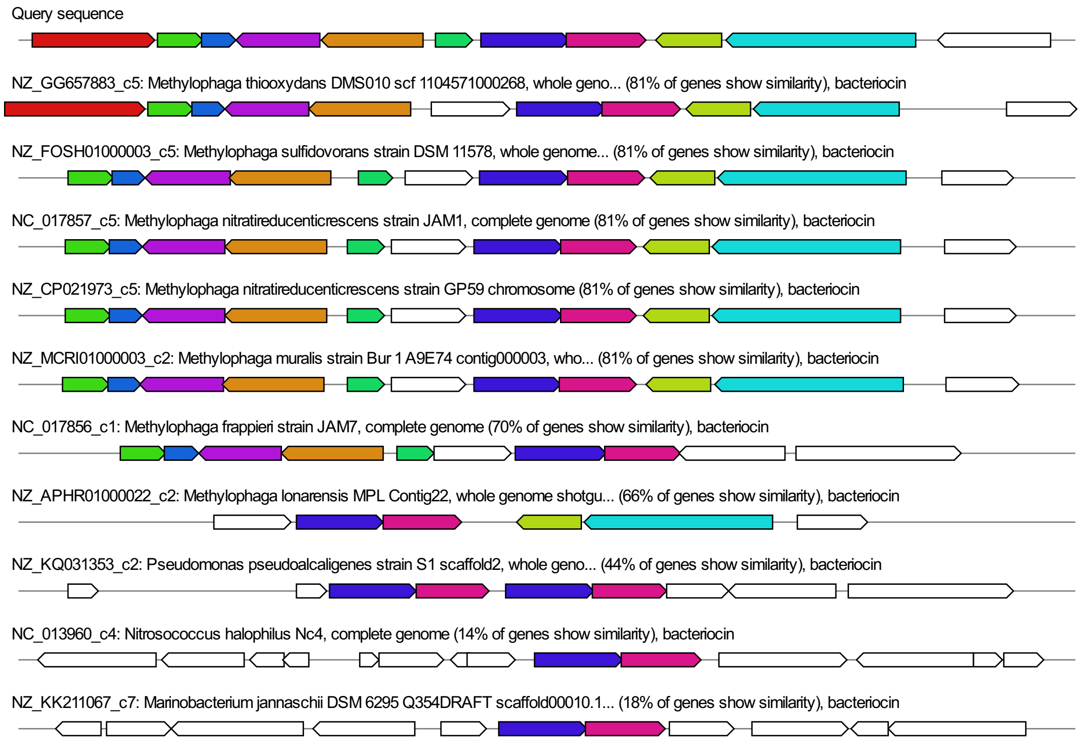

Figure S13 – AntiSMASH clusterblast output from a bacteriocin recovered from MAG Bin_60_1.

Table S10 - Distribution of the 10 KS domain OTUs recovered from the metagenomes matching with higher identity to MiBIG database.

Sample	OTU ID	BGC_acession	MiBiG product	Identity (%)	Query cover (%)	evalue
**YP_20**	out_dom_div_8_bf_NODE_12_length_387_cov_1_472892_1_387_-_1-97	BGC0000864	eicosapentaenoic acid-like compound	82.292	100	1.75e-53
**NB_250**	out_dom_div_3_bf_NODE_1_length_433_cov_1_034392_1_433_-_1-49	BGC0000284	phenolic lipids	74.419	90	1.98e-19
**TR_50**	out_dom_div_5_bf_NODE_4_length_472_cov_1_179856_1_472_-_15-110	BGC0000862	eicosapentaenoic acid	72.632	98	2.96e-45
**TR_250**	out_dom_div_6_bf_NODE_14_length_398_cov_1_058309_1_398_-_9-132	BGC0001051	thuggacin	71.545	100	2.77e-51
**YP_20**	out_dom_div_8_bf_NODE_5_length_473_cov_1_007177_1_473_-_14-156	BGC0001029	nostophycin	71.429	99	4.78e-64
**NB_50**	out_dom_div_2_bf_NODE_12_length_402_cov_1_158501_1_402_+_8-133	BGC0001165	curacin	71.2	100	1.45e-56
**YP_20**	out_dom_div_8_bf_NODE_20_length_352_cov_1_491582_1_352_+_0-79	BGC0000862	eicosapentaenoic acid	70.37	100	5.66e-37
**TR_250**	out_dom_div_6_bf_NODE_21_length_356_cov_1_627907_1_356_+_0-84	BGC0000862	eicosapentaenoic acid	69.767	100	9.3e-40
**NB_5**	out_dom_div_1_bf_NODE_12_length_398_cov_1_145773_1_398_-_0-132	BGC0001630	kasumigamide	69.697	100	3.3e-58
**NB_5**	out_dom_div_1_bf_NODE_4_length_472_cov_0_988010_1_472_+_0-156	BGC0001125	puwainaphycins	69.231	100	2.15e-68
